# Transcriptome analysis reveals significant differences between primary plasma cell leukemia and multiple myeloma even when sharing a similar genetic background

**DOI:** 10.1038/s41408-019-0253-1

**Published:** 2019-11-20

**Authors:** Elizabeta A. Rojas, Luis A. Corchete, María Victoria Mateos, Ramón García-Sanz, Irena Misiewicz-Krzeminska, Norma C. Gutiérrez

**Affiliations:** 1 Cancer Research Center-IBMCC (USAL-CSIC), Salamanca, Spain; 2 Institute of Biomedical Research of Salamanca (IBSAL), Salamanca, Spain; 3 Hematology Department, University Hospital of Salamanca, Salamanca, Spain; 4 Centro de Investigación Biomédica en Red de Cáncer (CIBERONC), CB16/12/00233, Salamanca, Spain; 5 National Medicines Institute, Warsaw, Poland

**Keywords:** Myeloma, Cancer genetics

## Abstract

Primary plasma cell leukemia (pPCL) is a highly aggressive plasma cell dyscrasia characterised by short remissions and very poor survival. Although the 17p deletion is associated with poor outcome and extramedullary disease in MM, its presence does not confer the degree of aggressiveness observed in pPCL. The comprehensive exploration of isoform expression and RNA splicing events may provide novel information about biological differences between the two diseases. Transcriptomic studies were carried out in nine newly diagnosed pPCL and ten MM samples, all of which harbored the 17p deletion. Unsupervised cluster analysis clearly distinguished pPCL from MM samples. In total 3584 genes and 20033 isoforms were found to be deregulated between pPCL and MM. There were 2727 significantly deregulated isoforms of non-differentially expressed genes. Strangely enough, significant differences were observed in the expression of spliceosomal machinery components between pPCL and MM, in respect of the gene, isoform and the alternative splicing events expression. In summary, transcriptome analysis revealed significant differences in the relative abundance of isoforms between pPCL and MM, even when they both had the 17p deletion. The mRNA processing pathway including RNA splicing machinery emerged as one of the most remarkable mechanisms underlying the biological differences between the two entities.

## Introduction

Plasma cell leukemia (PCL) is an uncommon and aggressive plasma cell dyscrasia, characterised by the presence of more than 20% of plasma cells (PCs) and an absolute number of ≥2 × 10^9^/L of PCs in peripheral blood^1,2^. PCL is classified as primary (pPCL) when detected de novo in patients with no evidence of previous multiple myeloma (MM), or as secondary (sPCL) in patients with relapsed or refractory MM that progresses to a leukemic phase^1,3,4^. Around 60% and 40% of PCLs are pPCL and sPCL, respectively^5^. pPCL accounts for less than 3% of all malignant plasma cell disorders^6–8^. As a consequence, the current knowledge regarding the molecular basis of pPCL is quite limited by the small number of cases included in most series.

Various studies have reported that pPCL patients show different clinical and biological features from those of MM. pPCL is associated with a dismal prognosis, whereby median survival is about 10 months. Extramedullary plasmacytomas, renal failure and massive bone marrow infiltration are more frequently observed in pPCL than in MM. Conversely, pPCL exhibits a lower prevalence of bone disease^1,2,9–11^.

Cytogenetic studies have shown that pPCL features elevated genomic instability, especially with respect to, karyotypic complexity, and higher prevalence of 17p13 deletions and 1q gains. Of the translocations involving the immunoglobulin heavy chain locus (*IGH*), t(11;14) and t(14;16) are more frequent in pPCL than in MM^11–14^. In recent years, the development of high-throughput technologies has given rise to a detailed knowledge about molecular characteristics of pPCL. Several studies have investigated the gene mutation patterns^15^, and differential gene and miRNAs expression profiles^16–20^, establishing differences and similarities between pPCL and sPCL, and MM.

Many of the genomic differences detected between pPCL and MM could be attributed to the dissimilar distribution of genetic abnormalities between the two entities^15^. For instance, the overrepresentation of 17p deletions in pPCL could explain some of the differences observed in the molecular and genomic patterns of pPCL and MM. However, a more unfavourable genetic background does not fully explain the ominous prognosis of this plasma cell neoplasm. In fact, although 17p deletion is associated with poor outcome and extramedullary disease in MM, the presence of this abnormality does not confer the degree of aggressiveness observed in pPCL.

We surmised that the comparison of the transcriptome profiles of pPCL and MM cases bearing similar cytogenetic abnormalities could provide valuable insights into new molecular mechanisms responsible for the different clinical outcomes of both diseases, and not reliant on particular chromosomal abnormalities. For this purpose, we analysed the transcriptome of pPCL and MM patients, using samples with 17p deletion and a similar cytogenetic profile, and focusing not only on gene expression profiling previously studied by other authors, but also on isoform expression and alternative splicing patterns.

## Materials and methods

For more specific information, see the *Online Supplementary File*.

### Patients and samples

Nine patients with newly diagnosed pPCL and ten with newly diagnosed MM were selected for the study based on the presence of 17p deletion. The median age of patients was 61 years (range: 54–89 years) for MM patients and 64 years (range: 45–83 years) for pPCL patients. The research ethics committee of the University Hospital of Salamanca approved the study in accordance with the Declaration of Helsinki principles.

Plasma cells were isolated from bone marrow samples using the AutoMACs immunomagnetic system (Miltenyi Biotec), as previously described^21^. Purity was greater than 95% in all MM and pPCL cases. Interphase fluorescence *in situ* hybridisation studies were performed as previously described^21^.

### Nucleic acid extraction, quantitative real-time PCR and *TP53* mutation analysis

RNA and DNA were extracted using AllPrep DNA/RNA Mini Kit (Qiagen). RNA integrity was assessed using Agilent 2100 Bioanalyzer. Total RNA (200 ng) was reverse-transcribed to cDNA using the SuperScript First-Strand Synthesis System (Thermo Fisher). Gene expression was evaluated with TaqMan (Applied Biosystems) qRT-PCR assays, and isoform and exon expression were evaluated using SYBR Green (Bio-Rad) qRT-PCR mRNA assays. *PGK1* and *18* *S* genes were used as endogenous controls for gene and isoform/exon expressions, respectively, and the relative expression was expressed as 2^−^^∆Ct^.

*TP53* mutation status was determined using 100 ng of genomic DNA by Sanger sequencing, as previously reported^22^.

### Human transcriptome arrays

Total RNA was amplified, labelled and hybridised to GeneChip® Human Transcriptome Array 2.0 from Affymetrix. Complete microarray data are available from the Gene Expression Omnibus under accession number GSE131216. Unsupervised multidimensional scaling (MDS) was performed using the Euclidean distance as the distance measure and the group average as the linkage method. Samples in the MDS from the CoMMpass dataset were classified in subgroups using model-based methods from the mclust R package.

Differential expression was analysed using the samr package (v2.0) in R. Gene and isoform level probesets with expression values less than the mean of the microarray antigenomic control expression values in all samples were excluded from further analysis. Genes and isoforms with a value of *q* < 0.05 were considered statistically significant and were selected for the KEGG pathway overrepresentation analysis in the Webgestalt suite^23^. Alternative splicing (AS) analysis was performed using the Affymetrix Transcriptome Analysis Console (TAC) version 4.0.1.36.

### Computational RNA-binding site prediction

The SpliceAid database was used to identify potential exonic splicing enhancer (ESE) motifs recognised by human serine/arginine-rich (SR) proteins^24^.

### Statistical analysis

The significance of differences in experiments was assessed by the two-sided Student’s *t* test for unpaired samples. Pearson correlation coefficients were calculated to measure associations between the results of HTA 2.0 and qRT-PCR assays. Fisher’s exact tests with two-tailed were calculated using the GraphPad QuickCalcs website: https://www.graphpad.com/quickcalcs/contingency1/. Values of *p* and *q* less than 0.05 were considered statistically significant. All statistical analyses were conducted using IBM SPSS Statistics 22.0 and the SIMFIT v7.0.9 package (Bardsley, University of Manchester, UK).

## Results

### Genetic abnormalities in pPCL and MM patients

All pPCL and MM samples carried the 17p deletion. A balanced distribution of other genetic abnormalities in the two entities was also taken into account when selecting the samples. Genetic abnormalities analysed by FISH are summarised in Table 1. The frequencies of *IGH* translocations according to 14q32 partners, chromosome 1 abnormalities and 13q deletions are shown in Table 1. Fisher’s exact test revealed no statistically significant differences between MM and pPCL (Table 1).

**Table 1:** Prevalence of genetic aberrations detected by FISH in pPCL and MM

	pPCL (*n* = 9)	MM (*n* = 10)	*p*-value (Fisher’s exact test)
**17p del**	9/9 (100%)	10/10 (100%)	1.00
**13q del**	7/9 (78%)	6/10 (60%)	0.63
**1q gain**	5/9 (56%)	4/10 (40%)	0.65
**1p del**	2/9 (22%)	1/10 (10%)	0.58
**t(11;14)**	3/9 (33%)	2/10 (20%)	0.63
**t(4;14)**	2/9 (33%)	0/10 (0%)	0.21
**t(14;16)**	1/9 (11%)	0/10 (0%)	0.47

To better characterise the *TP53* status, mutations were analysed in all patients with an available genomic DNA sample (18/19 samples). *TP53* mutations were identified in four of eight pPCLs (50%) and three of ten MM (30%) (*p* = 0.63, Fisher’s exact test). Six of the seven identified mutations were missense substitutions (86%), and were localised in the DNA binding domain, distributed between exons 5 and 8. The mutated amino acid residues were in areas with hotspot properties and direct DNA contact site. According to their functionality as transcription factor the missense mutations detected in the seven samples predicted an inactive p53 (Supplementary Table 1).

### Differentially expressed genes between pPCL and MM

To investigate the transcriptome signature of pPCL and MM patients, Human Transcriptome Array 2.0 analysis was performed. These microarrays allow us to explore gene expression profile, as well as splicing events and isoform expression, using millions of probes that cover exon–exon splice junctions, and coding and non-coding isoforms.

**Fig. 1:**
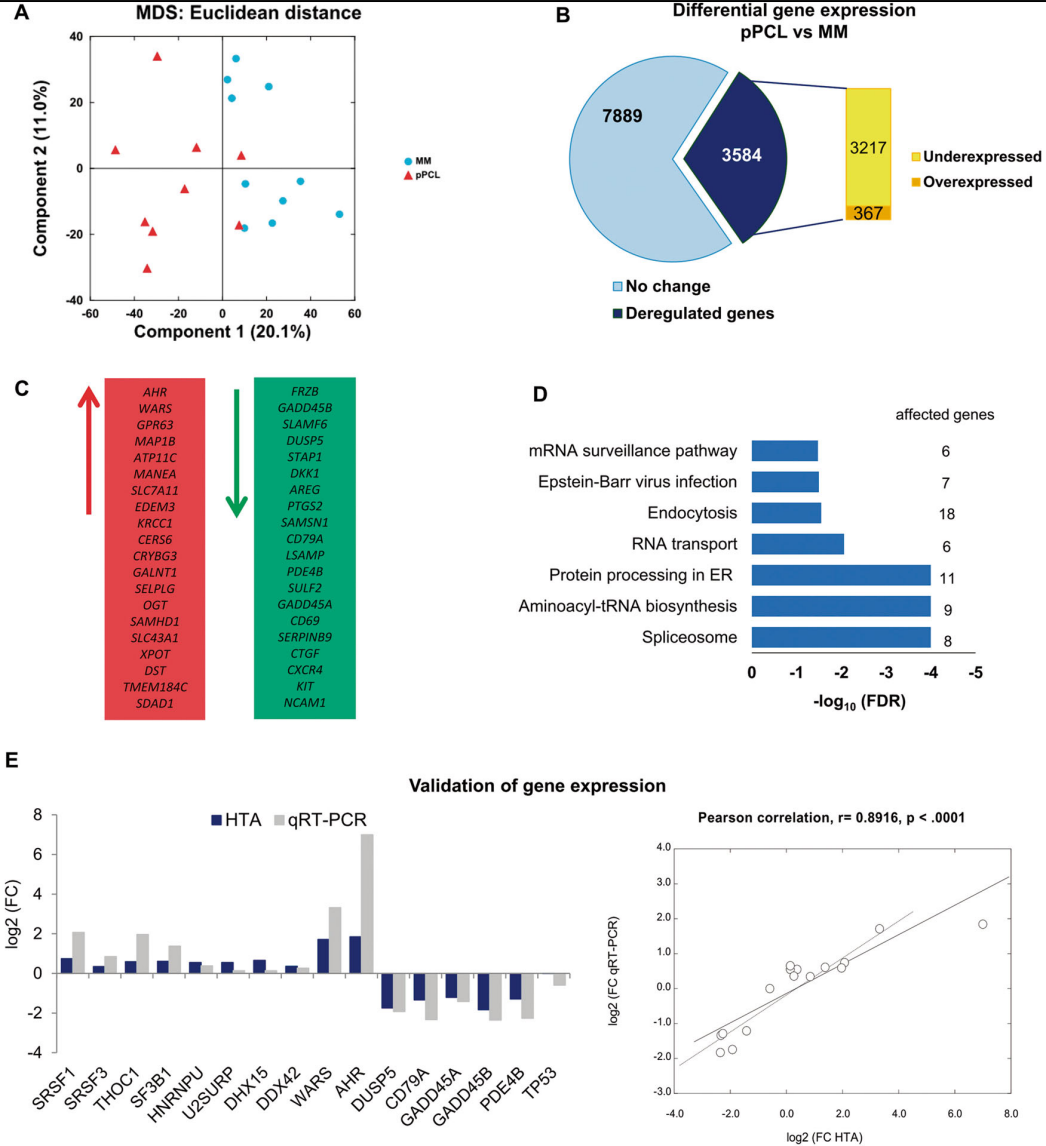
Gene expression analysis. **a** Unsupervised analysis of gene expression in MM and pPCL samples using multidimensional scaling (MDS) of 19 samples (9 pPCL and 10 MM) based on the expression of 35,345 genes. Samples appear to be clustered in two groups, based on the Euclidean distance. **b** Differential gene expression of pPCL relative to MM samples using the samr package in R. Genes with positive log_2_ FC and *p* < 0.05 were considered overexpressed, and genes with negative log_2_ FC and *p* < 0.05 were considered underexpressed, in pPCL compared with MM samples. **c** List of the 40 most deregulated genes: the 20 most overexpressed and the 20 most underexpressed genes in pPCL relative to MM samples. **d** Pathway enrichment analysis at the gene level using Webgestalt suite. The number of affected genes in each pathway is shown. The statistical significance of the enrichment is expressed as -log_10_ FDR using the Benjamini–Hochberg method. **e** Experimental validation of gene expression of HTA microarray results. The mRNA levels of genes were assessed by qRT-PCR. The results are shown as the magnitude of change (log_2_ FC) between pPCL and MM samples, using *18* *S* rRNA as the endogenous control gene. The Pearson correlations of gene expression measured by qRT-PCR and by HTAs in pPCL (*n* = 9) and MM (*n* = 10) samples are also shown.

First, we explored the distribution of the samples in clusters based on similarities in gene expression data using unsupervised multidimensional scaling (MDS). The first component in this analysis clearly separated MM from pPCL samples (Fig. 1a), although two pPCLs were closer to MM samples. We asked whether different clusters could also be identified across a large series of MM cases with del17p. We used RNA-Seq data from 73 MM patients with del17p from the MMRF CoMMpass trial (NCT01454297) to address this question. We found no clusters, but samples were apparently randomly distributed (Supplementary Fig. 1).

Gene expression analysis from HTAs identified a total of 3584 genes differentially expressed between the pPCL and MM groups, of which 367 were overexpressed and 3217 genes were underexpressed in pPCL relative to MM patients (Fig. 1b). The list of the 40 genes with the greatest and least fold change (FC) is shown in Fig. 1c. Functional analysis using the KEGG pathway database revealed statistically significant enrichment for spliceosome, aminoacyl-tRNA biosynthesis, protein processing in endoplasmic reticulum (ER) and RNA transport (Fig. 1d). The heatmap, using only the genes from overrepresented KEGG pathways, distinguished two entities with different gene expression patterns and with a clear prevalence of underexpression in pPCL compared with MM (Supplementary Fig. 2).

Results of gene expression profiling were validated by qRT-PCR. We chose 15 genes, eight components of the spliceosome selected from the functional analysis using KEGG database (*SRSF1, SRSF3, SF3B1, DDX42, DHX15, HNRNPU, THOC1*, and *U2SURP*), and seven genes from the list of the 40 most deregulated genes, whose role in cancer is less well understood (*WARS, AHR, DUSP5, CD79A, GADD45A, GADD45B*, and *PDE4B*). *TP53* gene expression was also quantified. The primers used in these assays are shown in Supplementary Table 2. As shown in Fig. 1e, we observed a high concurrence in the FC direction between HTA and qRT-PCR analysis for all the 16 genes considered. These results also revealed a strong positive and statistically significant correlation (*r* = 0.89, *p* < 0.0001) between the HTA microarray and qRT-PCR results (Fig. 1e).

### Differentially alternative splicing events in pPCL and MM

**Fig. 2:**
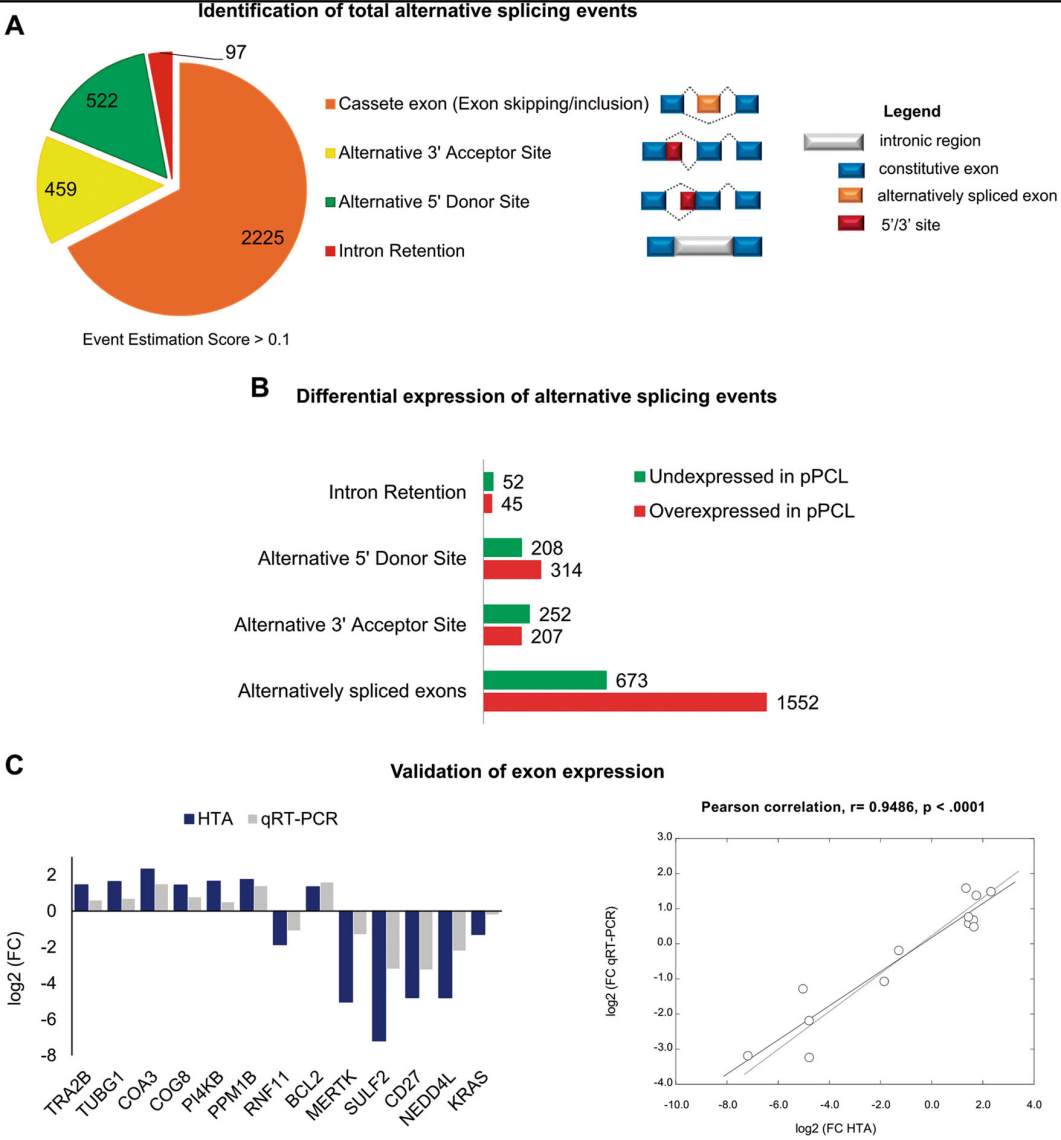
Alternative splicing analysis. **a** Analysis of statistically significant alternative splicing events irrespective of gene expression changes between pPCL and MM samples. ASEs were identified using Affymetrix TAC software and classified into four main types of pattern: cassette exon (inclusion or exclusion of exons), alternative 5′ donor site, alternative 3′ acceptor site, and retained introns. Only the events with an event estimation score > 0.1 and an ASE assigned according an absolute splicing index of 2 and exon *p* < 0.05 were considered. **b** Differential ASEs expression of pPCL compared with MM samples using the TAC. ASEs with positive log_2_ FC and *p* < 0.05 were considered as overexpressed, and ASEs with negative log_2_ FC and *p* < 0.05 were considered as underexpressed, in pPCL compared to MM samples. **c** Experimental validation of exon expression of HTA microarray results. The results are shown as the magnitude of change (log_2_ FC) between pPCL and MM samples, using *PGK1* as the endogenous control gene. The Pearson correlation of exon expression measured by qRT-PCR and by HTAs in pPCL (*n* = 9) and MM (*n* = 10) samples are also shown.

Next, we aimed to identify alternative splicing events (ASEs) differentially expressed between pPCL and MM patients. We detected 2873 genes affected by at least one ASE, and the expression of 3303 splicing events (Fig. 2a). The most abundant ASEs were the cassette exon, with 2225 exons included or excluded from mRNAs, followed by alternative 5′ donor site, alternative 3′ acceptor site, and intron retention (Fig. 2a). The differential expression of these four types of ASEs in pPCL relative to MM is shown in Fig. 2b. In this regard, among all ASEs we found a large number of alternatively spliced exons differentially expressed between the two groups (Fig. 2b). KEGG pathway analysis of genes affected by deregulation of alternatively spliced exons, revealed that FoxO signalling, RNA transport, glucagon signalling, phospholipase D signalling and Epstein–Barr virus (EBV) infection pathways were the most significantly enriched (Supplementary Fig. 3).

We validated the differential expression of the cassette exon event of 13 selected genes (*TRA2B, TUBG1, BCL2, COA3, COG8, PI4KB, PPM1B, RNF11, MERTK, SULF2, CD27, KRAS* and *NEDD4L*) by qRT-PCR (Supplementary Table 3). The selection criteria were statistically significant exons (*p* < 0.05) with the highest and lowest FC between pPCL and MM, and longer than 70 nucleotides. The primers used for these assays are shown in Supplementary Table 4. Thus, the downregulation of exons of *SULF2, CD27, NEDDL4, RNF11, MERTK* and *KRAS* genes, and the upregulation of exons of *TRA2B, TUBG1, BCL2, COA3, COG8, PI4KB*, and *PPM1B* genes observed in the HTA microarrays of pPCL were validated by qRT-PCR (Supplementary Fig. 4). Furthermore, the Pearson correlation analysis revealed a strong, positive and statistically significant correlation (*r* = 0.9488, *p* < 0.0001) between HTA microarrays and qRT-PCR results of the 13 selected alternatively spliced exons (Fig. 2c).

It is well known that changes in the expression of different spliceosome genes can affect alternative splicing profile of cancer cells^25–27^. In particular, some of the components of the SR protein family have been shown to be upregulated in various cancers, in which regulate the splicing of many genes. These data strongly emphasise the important role of alternative splicing in tumorigenesis^28,29^. Therefore, we investigated whether the deregulated gene expression of spliceosome components found in pPCL as compared to MM, was correlated with the occurrence of deregulated splicing events detected in this study. We focused on *SRSF1* and *SRSF3* genes, whose overexpression in pPCL was validated by qRT-PCR (as shown in Fig. 1e and Supplementary Fig. 5A). A high correlation between *SRSF1* and *SRSF3* was observed (Supplementary Fig. 5A), which is noteworthy because the expression of *SRSF1* and *SRSF3* genes is mutually regulated and coexpressed in normal and tumour cells, and *SRSF3* is able to regulate the alternative splicing of *SRSF1* gene^30^.

We next explored the correlation between *SRSF1* and *SRSF3* genes with the 13 validated exons (Fig. 5b) alternatively spliced and differentially expressed between pPCL and MM (*TRA2B, TUBG1, BCL2, COA3, COG8, PI4KB, PPM1B, RNF11, MERTK, SULF2, CD27, KRAS*, and *NEDD4L*). We found a negative significant correlation between *SRSF1* and the expression of *CD27* exon 2 (*r* = −0.71, *p* = 0.0006), *NEDD4L* exon 3 (*r* = −0.55, *p* = 0.0130) and *RNF11* exon 3 (*r* = −0.4755, *p* = 0.0396) (Supplementary Fig. 5B, C). In the case of *SRSF3*, a negative significant correlation with *CD27* exon 2 (*r* = −0.66, *p* = 0.0019) and *MERTK* exon 3 (*r* = −0.5587, *p* *=* 0.0129) was also detected (Supplementary Fig. 5B, D).

These results indicate that the alternative splicing of *CD27*, *NEDD4L*, and *RNF11* genes could be regulated by the splicing factor *SRSF1*, and the alternative splicing of *MERTK* and *CD27* genes could be controlled by *SRSF3*.

### Differentially expressed isoforms between pPCL and MM

**Fig. 3:**
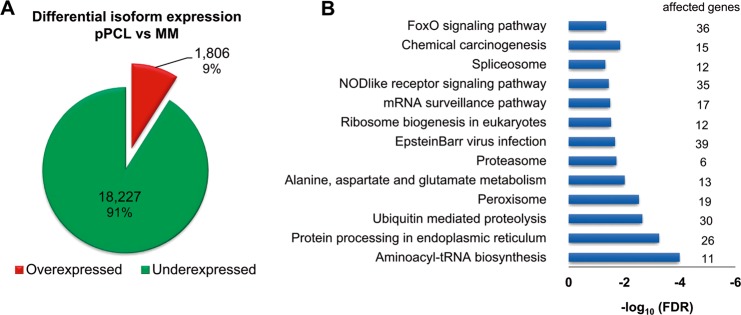
Differential isoform expression between pPCL and MM samples. **a** Isoforms with positive log_2_ FC and *p* < 0.05 were considered overexpressed, and isoforms with negative log_2_ FC and *p* < 0.05 were considered underexpressed. **b** KEGG enrichment analysis based on genes with deregulated isoforms. The statistical significance of the enrichment analysis is expressed as −log_10_ FDR using the Benjamini–Hochberg method.

Alternative splicing, carried out by the spliceosome machinery, leads to mRNA isoforms that encode different proteins. Since we had observed differential expression of ASEs between MM and pPCL, we searched for differences in mRNA isoform expression. We identified 20033 deregulated isoforms associated with 6254 gene IDs curated by the HGNC (Hugo Gene Nomenclature Committee), of which 1806 isoforms were overexpressed and 18227 were underexpressed in pPCL patients relative to MM patients (Fig. 3a). The enrichment analysis identified EBV infection, spliceosome and proteasome pathways, among others, as statistically significantly overrepresented KEGG pathways (Fig. 3b). In the unsupervised analysis based on isoform expression, the samples tended to be separated into two groups, as was the case with gene expression analysis (Supplementary Fig. 6).

**Fig. 4:**
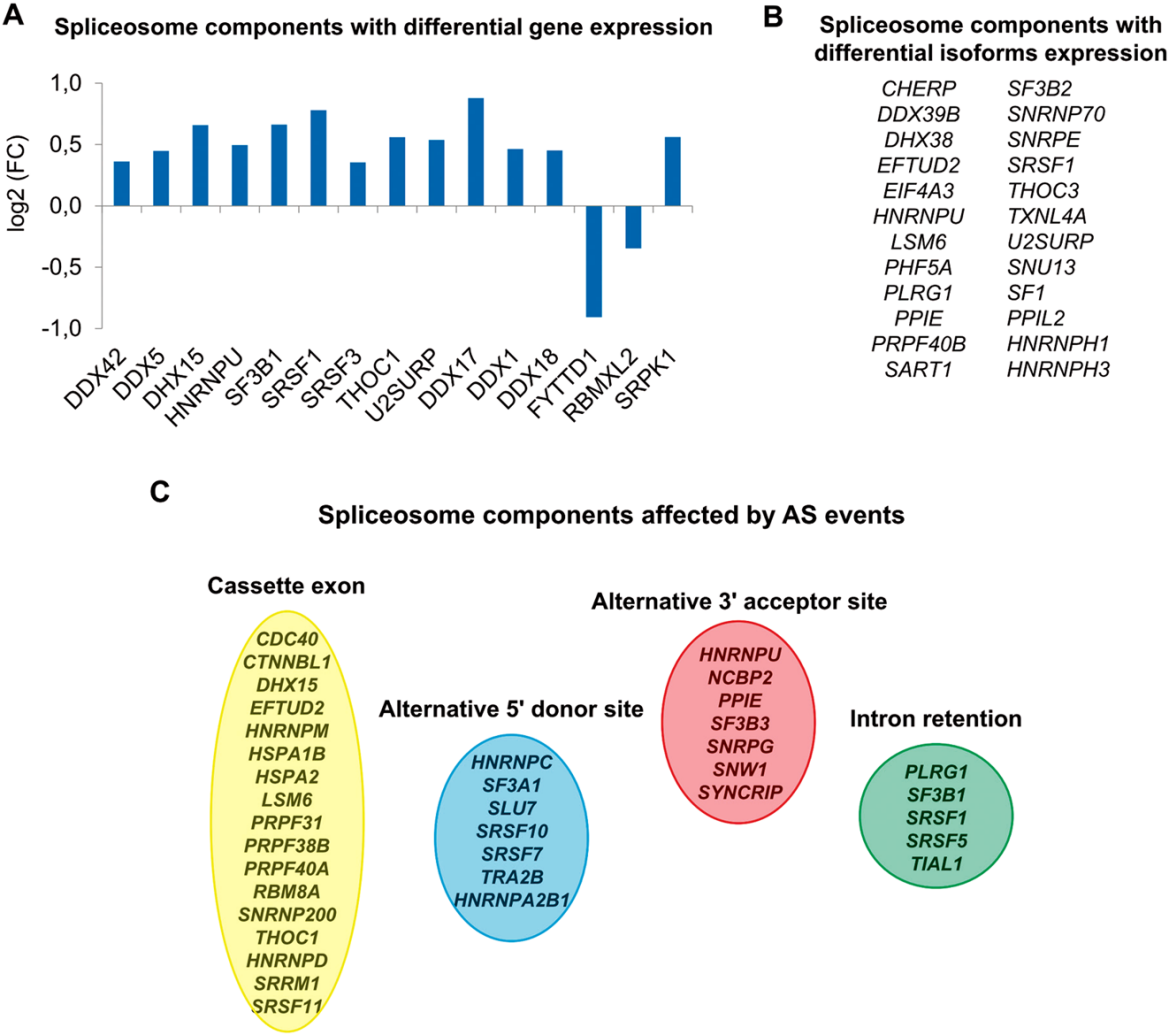
Deregulation of the spliceosomal machinery between pPCL and MM. Spliceosomal components with differential gene expression (*q* < 0.05) **a** with differential isoform expression (*q* < 0.05) **b** and those affected by alternative splicing events (*q* < 0.05) **c**.

It should be pointed out that across the entire transcriptome analysis we found significant differences in the expression of the components of the spliceosomal machinery between pPCL and MM. We found 15 spliceosome genes differentially expressed between the two groups. Moreover, 24 spliceosome components displayed differential expression of some of their isoforms, and 36 were affected by AS events (Fig. 4).

**Fig. 5:**
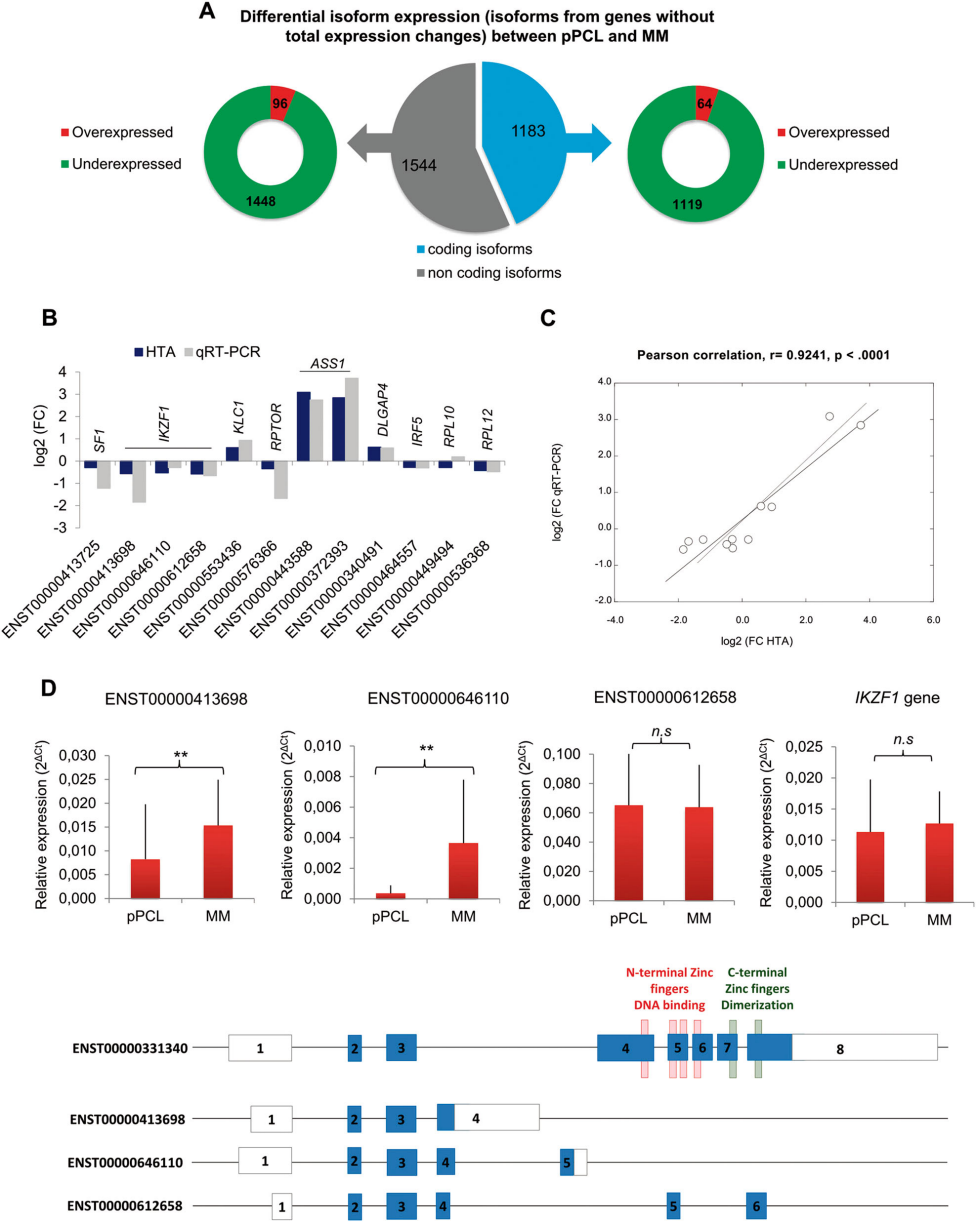
Identification of differentially expressed isoforms of genes without total expression changes. **a** Classification of deregulated isoforms according to coding (in blue) and non-coding (in grey) isoforms among the non-differentially expressed genes. The right side shows the coding isoforms that were overexpressed and underexpressed in pPCL relative to MM. The left side shows the non-coding isoforms that were overexpressed and underexpressed in pPCL relative to MM. For both, coding and non-coding isoforms with positive log_2_ FC and *p* < 0.05 were considered overexpressed, and isoforms with negative log_2_ FC and *p* < 0.05 were considered underexpressed. Genes with a value of *p* > 0.05 were considered not to be differentially expressed. **b** Experimental validation of differential isoform expression from genes without total expression changes in the HTA microarrays. The mRNA levels of isoforms (Ensembl ID) were assessed by qRT-PCR. The results are shown as the log_2_ FC between pPCL and MM samples, using the *PGK1* gene as the endogenous control. The expression difference of ten isoforms between pPCL and MM was statistically significant, and the expression difference of ENST00000449494 (*RPL10* isoform) and ENST00000612658 (*IKZF1* isoform) between pPCL and MM were not statistically significant. **c** Pearson correlation of isoform expression measured by qRT-PCR and by HTAs in pPCL (*n* = 9) and MM (*n* = 10) samples. **d** *IKZF1* isoform analysis. The mRNA levels of ENST00000413698, ENST00000646110, ENST00000612658 and *IKZF1* gene were assessed by qRT-PCR. The results are shown as the relative expression (calculated as 2^−^^ΔCt^) ± s.d between pPCL and MM samples, using *PGK1* as endogenous control gene. Statistically significant differences between pPCL and MM samples are represented as **p < 0.01, *p < 0.05 and *n.s*. non-significant (unpaired two-sided *Student’s t* test). Schematic representation of the three downregulated *IKZF1* coding isoforms analysed by qRT-PCR in pPCL compared with MM samples is provided below. The longest isoform (ENST00000331340, NM_006060) contains four N-terminal zinc finger motifs (red), and two C-terminal zinc fingers (green). Exons are numbered from 1 to 8. Coding exons are represented by blue boxes and UTR regions by black boxes.

In recent years, some studies have reported the advantage of the analysis of isoform expression pattern compared with standard gene expression profiling in cancer research^31,32^. Therefore, we also focused on those expressed genes (selection criteria for “expressed genes” in Materials and Methods section) that were not differentially expressed, but whose isoforms were differentially expressed. We found 2727 deregulated isoforms belonging to 1249 expressed genes, 1183 of which were coding isoforms (Fig. 5a). A prevalence of underexpressed isoforms in pPCL patients relative to MM was also found.

To validate the differential isoform expression detected by HTA, we focused on the coding isoforms, of which 64 were overexpressed and 1119 underexpressed in pPCL compared to MM patients (Supplementary Fig. 7A). We selected 12 coding isoforms differentially expressed (*q* < 0.05) and distributed according to high, medium and low FC, to be validated by RT-PCR (Supplementary Fig. 7A). In the case of genes with two or three deregulated isoforms a specific sequence was needed in each isoform for primer design. The selected isoforms and the primers used are shown in Supplementary Tables 5 and 6, respectively.

The differential expression of ten of the twelve isoforms tested by qRT-PCR was consistent with the results obtained by HTA (Fig. 5b). Subtle discrepancies were observed for isoforms ENST00000449494 (*RPL10* isoform) and ENST00000612658 (*IKZF1* isoform). A strong, positive and statistically significant correlation (*r* = 0.92, *p* < 0.0001) between the two methods was observed (Fig. 5c).

The overexpressed isoforms ENST00000553436 (*KLC1* gene), ENST00000443588 and ENST00000372393 (*ASS1* gene), and ENST00000340491 (*DLGAP4* gene) are isoforms coding for protein domains, which should be biologically effective and able to exert their function. On the other hand, the isoforms ENST00000413698 and ENST00000646110 (*IKZF1* gene), ENST00000576366 (*RPTOR* gene) and ENST00000413725 (*SF1* gene) are among the underexpressed isoforms, which include regions coding for protein domains whose lack probably lead to loss of some of the protein functions. We were struck in particular by the *IKZF1* gene (Ikaros protein), which was among the genes containing various differentially expressed isoforms. The *IKZF1* gene is composed of eight exons and produces 23 splice variants by alternative splicing, which are differentiated by alternative use of exons and subsequently in their functional domain composition, with distinct biological functions (Supplementary Fig. 6B). Six *IKZF1* isoforms were underexpressed (*q* < 0.05) in the HTA analysis comparing pPCL and MM (Supplementary Fig. 7C). Among the four protein coding isoforms, three could be quantified by qRT-PCR, considering the specific sequences in each isoform. The coding isoforms ENST00000413698 and ENST00000646110 were significantly underexpressed in pPCL (*p* < 0.01) as revealed by qRT-PCR, while the *IKZF1* gene expression was equally expressed in both plasma cell dyscrasias, corroborating the HTA results (Fig. 5d). However, we found similar expression of the isoform ENST00000612658 in pPCL and MM (Fig. 5d).

### Identification of potential exonic splicing enhancers

Alternative splicing produces different isoforms from a single gene, contributing significantly to proteomic diversity. This process is mediated by the splicing machinery, the spliceosome, which comprises more than 300 splicing factors that bind to specific sequence elements in the pre-mRNA sequence. Among the splice site signals in this sequence, exonic splicing enhancers (ESEs) are short and degenerative sequences that enhance splice-site recognition in constitutively and alternatively spliced exons^33^. ESEs act as binding sites for SR RNA-binding proteins, a family of conserved splicing factors that participate in multiple steps of the spliceosome assembly, during RNA splicing^34^. A broad range of sequences can function as ESEs in a particular context, and distinct SR proteins have different ESE specificities. The development of several resources for identifying ESEs binding sites and the global deregulation of the spliceosomal machinery prompted us to use one of these databases^24^ to look for potential ESE motifs recognised by human SR proteins in our transcriptome dataset.

**Fig. 6:**
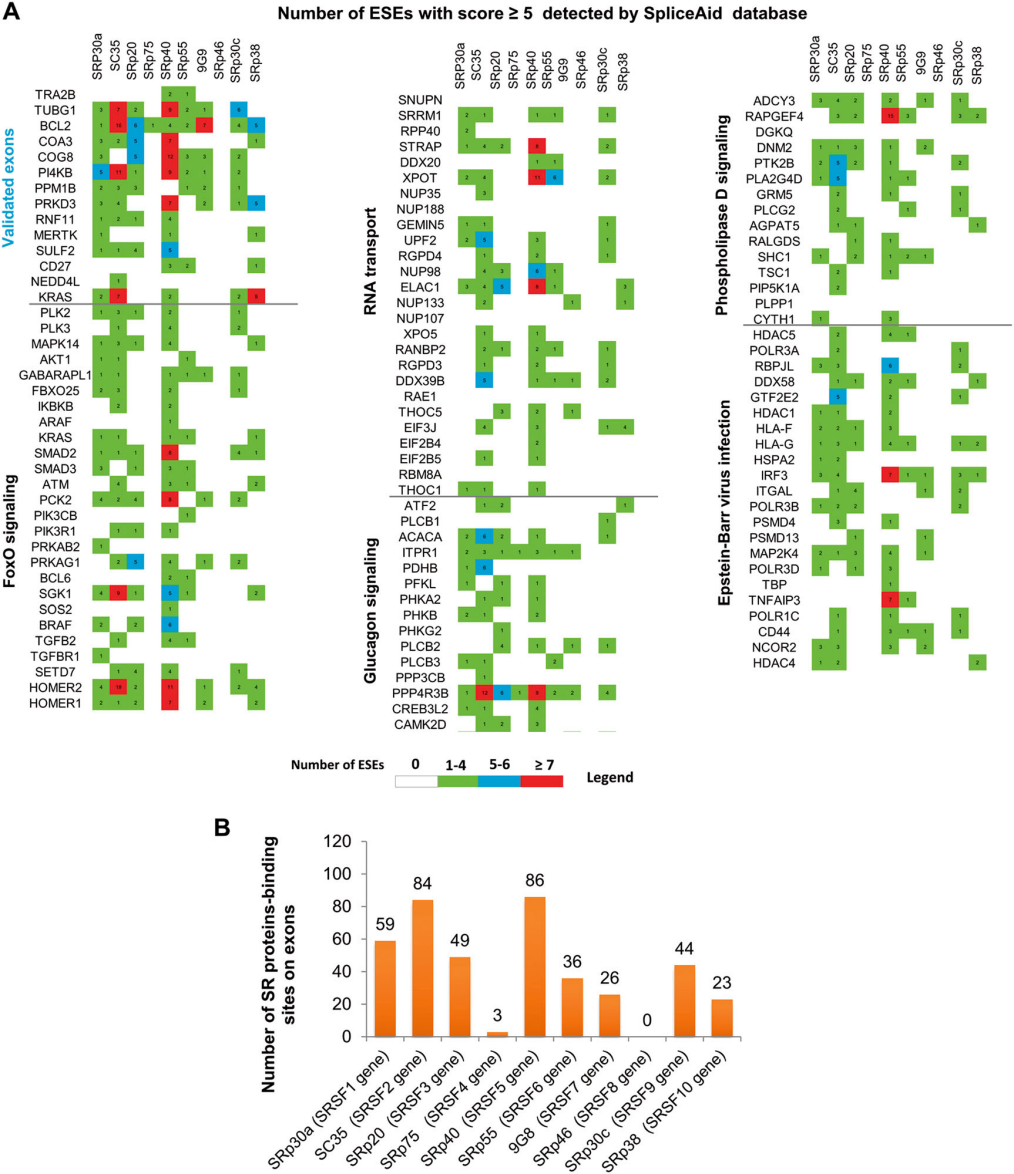
Predictive identification of exonic splicing enhancers using the SpliceAid database. **a** Detection of ESEs in the 13 experimentally validated exons with differential expression between pPCL and MM, and on 104 exons with differential expression detected by KEGG pathway analysis affected by inclusion or exclusion of exons. Only the ESEs with a score of ≥5 assigned by the SpliceAid database were considered. The legend refers to the number of ESEs detected per exon for each SR protein. **b** Number of SR protein-binding sites in the 117 exons analysed.

First, we examined exons differentially expressed between pPCL and MM that were validated by qRT-PCR assays. We only considered ESEs with a score of ≥ 5 in the SpliceAid database. The prediction algorithm used identified a very large number of ESEs. The analysis of the 13 exon sequences (*TRA2B, TUBG1, BCL2, COA3, COG8, PI4KB, PPM1B, RNF11, MERTK, SULF2, CD27, KRAS*, and *NEDD4L* genes) based on the SpliceAid database, predicted that all of them had at least one binding site for SR proteins (Fig. 6a). SRp40, SRp30a and SC35 proteins had the highest number of binding sites on the 13 exons, while SRp75 and SRp46 SR proteins had one and zero, respectively (Fig. 6a). These results indicate a possible strong effect of SRp40, SRp30a and SC35 proteins of all the SR proteins involved in the splicing of those pre-mRNAs (with a greater number of binding sites). In fact, SR proteins were overexpressed at the gene level in pPCLs compared with MM patients, although the differences were only statistically significant for *SRSF1* and *SFSF3* (Supplementary Fig. 8).

Secondly, we analysed 104 exons differentially expressed between pPCL and MM that were detected in KEGG pathway analysis of genes affected by inclusion or exclusion of exons (Supplementary Table 6). More than 70% of exons had binding sites for SRp40 and SC35 proteins, followed by SRp30a and SRp20, with 50% and 42%, respectively (Fig. 6b).

Since ESEs can be highly heterogeneous, their composition probably influences the occurrence of pre-mRNA splicing and thereby affects the transcriptome of cells. The higher number of ESEs found using the database indicates that prediction analysis needs to be validated experimentally to identify those that genuinely act by binding to SR proteins in the specific context of pPCL and MM.

## Discussion

In this study, we compared the transcriptome profiles of pPCL and MM using samples that shared 17p deletion and a similar pattern of other cytogenetic alterations. These selection criteria allowed us to investigate whether molecular mechanisms other than those associated with the simple predominance of a particular chromosomal abnormality could contribute to determine the dramatic aggressive outcome of pPCL. Using HTA microarrays we were able not only to study the gene expression pattern, as other authors have done, but also to investigate the isoform expression and the impact of alternative splicing on the pathology of these diseases.

In spite of the similar distribution of cytogenetic alterations in the two groups, the unsupervised analysis based on gene expression data distinguished two differentiated clusters. In contrast, the same kind of analysis using RNA-Seq data from the CoMMpass study did not identify any cluster in the set of MM patients with the 17p deletion. These results suggest that tumour PCs of pPCL have a significantly different transcriptome profile from that observed in the tumour PCs of MM, even though both diseases share the same tumour cells and have similar cytogenetic abnormalities. Indeed, we found a large number of genes that are differentially expressed between pPCL and MM, most of which are underexpressed in pPCL patients. Our results are not comparable with those of previous studies of gene expression profiling in pPCL and MM patients, since we selected patients with a similar genetic background across the two plasma cell dyscrasias^17^. This probably explains why the gene expression-based pathway enrichment analysis showed seven affected pathways, including the spliceosome, aminoacyl-tRNA biosynthesis and EBV infection, which have not been described before. Nevertheless, *SLAMF6, SULF2, STAP1*, and *AHR* genes, four of the 40 most significantly deregulated genes in pPCL were among the list of 503 genes reported by Todoerti et al.^17^ as being differentially expressed between the two groups.

Interestingly, six of the 20 most underexpressed genes founded in pPCL (*FRZB, DKK1, KIT, NCAM1, CTGF*, and *CXCR4*) are commonly associated with the bone marrow microenvironment and bone disease in MM. For example, the expression of the WNT inhibitors, *FRZB* and *DKK1* genes, is associated with osteolytic bone lesions in myeloma patients^35^. In fact, ~80% of MM patients develop osteolytic bone lesions, while in pPCL bone lesions are less frequent^36^.

*WARS* (tryptophanyl-tRNA synthetase) and *AHR* (aryl hydrocarbon receptor) genes were the two most overexpressed genes in pPCL. *WARS* is a housekeeping enzyme that participates in the protein synthesis, catalysing the specific ligation of tRNA with tryptophan^37^. The upregulation of *WARS* has been reported to be included in a high-risk transcriptional signature for pPCL correlated with shorter overall survival^17^. Remarkably, a recent study has demonstrated a novel non-canonical function of *WARS* in antiviral defence, so it is rapidly secreted in response to viral infection, leading to the *in vitro* and *in vivo* inhibition of virus replication^38^. Previous studies have reported high levels of expression of *WARS* in cells infected with human cytomegalovirus, hepatitis B virus, and even in mouse intestines infected with *Cholera vibrio*^39–41^. In our study, an unexpected finding was the deregulation of the EBV infection pathway as revealed by the KEGG analysis for gene, isoform and alternatively spliced exon expressions. In addition, a few studies have reported the use of cellular spliceosome machinery by EBV for the transcription of viral proteins in EBV-infected cells with splicing components^42,43^. We can speculate that the infection with EBV might lead to the overexpression of the *WARS* gene as an antiviral defence of the EBV-infected cells. However, the potential role of this response in the pathogenesis of pPCL is unknown. The *AHR* gene is a cytosolic ligand-activated transcription factor considered as a chemical sensor of xenobiotic-induced carcinogenesis^44^. Several studies have demonstrated that *AHR* is overexpressed and constitutively active, even in the absence of environmental ligands in a range of human tumours, such as cutaneous squamous cell carcinomas^45^, breast cancer^46^, adult T-cell leukemia^47^, pancreatic cancer^48^, and human glioma^49^. Recently, *AHR* levels have been inversely associated with survival of MM patients, indicating that *AHR* inhibition may be an attractive therapeutic option for the treatment of this disease^50^.

We also investigated alternative splicing events and isoforms expression in pPCL and MM patients. More than 2000 events of alternative splicing, predominantly inclusion or exclusion of exons, were detected. The use of a transcriptome array allowed us to identify more than 1000 deregulated coding isoforms, mostly underexpressed in pPCL, belonging to 658 genes whose expression did not significantly differ between pPCL and MM. Of these, the *SF1* (Splicing Factor 1) gene is a RNA-binding component of the spliceosomal machinery required for the ATP-dependent first step of spliceosome assembly. The deregulated *SF1* isoform is shorter than the canonical one and lacks the essential protein domain expression involved in their RNA binding functions. In this way, the *SF1* isoform is probably unable of efficiently exert its canonical function in spliceosome assembly, irrespective of *SF1* gene expression. We also found two deregulated *ASS1* (Argininosuccinate Synthase 1) isoforms that differs in the number of protein domains and features. The Ass1 protein is one of the enzymes of the urea cycle and catalyses the penultimate step of the arginine biosynthesis pathway. Thereby, *ASS1* gene is critical for the growth of human cancers, due to the role of L-arginine in different aspects of tumour metabolism and the immune system^51,52^. We can reasonably speculate that the overexpression of these two *ASS1* isoforms in pPCL patients could be associated with an increased proliferation of the PCs.

On the other hand, we detected six underexpressed *IKZF1* isoforms in pPCL patients in the HTA data, two of which were validated by qRT-PCR. Ikaros, encoded by the *IKZF1* gene, is a zinc-finger protein essential for lymphocyte development and which is important in MM pathology^53^. Their proteasomal degradation is a critical event in the mechanism of action of immunomodulatory drugs (IMiDs) against MM^54^. The 23 *IKZF1* isoforms produced by alternative splicing are differentiated by the alternative use of exons and subsequently in their functional domain composition, with distinct biological functions. However, only the isoforms that contain at least three of four N-terminal zinc fingers are capable of binding to DNA efficiently, and exert their function as a transcription factor. Nevertheless, the isoforms that contain the C-terminal zinc fingers that are responsible for dimerisation with other Ikaros isoforms and other family members have dominant negative functions by which they interfere with the DNA-binding functions of those proteins, including the inhibition of the activity of the full-length Ikaros protein^55–57^. Interestingly, in this study the two validated *IKZF1* isoforms are shorter than the canonical isoform and are non-DNA-binding isoforms with a distinct C-terminal compared with the canonical isoform. Likewise, we hypothesised that these two *IKZF1* non-DNA-binding isoforms could also bind to the other members of the zinc-finger protein family through the C-terminal domain and exert a dominant-negative effect by interfering with the activity of those DNA-binding isoforms and, consequently, transcription would not be activated^56,58^.

Overall, our results indicate that pPCL is a unique biological entity, and therefore different from MM. Even comparing MM and pPCL cases that share similar cytogenetic alterations, the transcriptome of pPCL is substantially and significantly different from that of MM. This work is the first of its kind to explore the expression of the spliceosomal components and the occurrence of alternative splicing events in pPCL compared with MM samples. Our findings also suggest the existence of a significant connection between alternative splicing deregulation and the molecular features of pPCL, and provide evidence of candidate genes and as yet unidentified pathways that could be pertinent to the biology of pPCL and that could contribute to the more aggressive clinical course of this neoplasm compared with that of MM. A limitation of this study is the small sample size related to the low incidence of pPCL. As such, further research is needed to validate the most relevant findings in other series of pPCL patients with del17p.

## Supplementary information

Online Supplementary File
