# Supplementary material for: Transcriptome analysis reveals significant differences between primary plasma cell leukemia and multiple myeloma even when sharing a similar genetic background
Source: Blood Cancer J. 2019 Nov 20;9(12):90. doi: 10.1038/s41408-019-0253-1 (PMC6868169; doi:10.1038/s41408-019-0253-1)
Supplement: Supplementary file 1 — Online Supplementary File [file 41408_2019_253_MOESM1_ESM.pdf]

Supplemental Methods

Quantitative real-time PCR

Specific primers on exons and specific regions of each isoform were designed. PCR conditions were 95^0 for 1 min (1 cycle); 95^0 for 45 s, 50-65^0 for 30 s and 72^0 for 1 min 30s (40 cycles), and finally 72^0 for 8 min. The different primers used, and their corresponding sequences are indicated in supplemental tables S2, S3 and S5.

Human transcriptome arrays analysis

Raw data were quantile normalized, background corrected and $\log_2$ transformed with the Robust Multi-array Average (RMA) algorithm using the oligo package (v.1.44.0) in R (v.3.5.0)[1]. BrainArray custom CDF files (version 19 Ensembl), at both gene and isoform level, were used as reference.

The heatmaps depicting the expression of the overrepresented genes in the KEGG pathways were constructed using a freely available web tool (http://www.heatmapper.ca)[2].

Alternative splicing (AS) analysis was performed using the Affymetrix Transcriptome Analysis Console (TAC) version 4.0.1.36. Raw data were normalized using the SST-RMA algorithm implemented in TAC. To identify AS events, we considered as statistically significant events those with an exon p-value < 0.05 and exon estimation score > 0.1. The event (exon) estimation score is a value from 0 to 1, where 1 represents a high likelihood for the alternative splicing event (ASE) selected from the Probe Selection Regions/Junction Probesets table provided by TAC, and represents how well the data fits into pre-defined splicing patterns.

Computational RNA-binding site prediction

SpliceAid is a database of human splicing factors and their experimentally evaluated target RNA binding sites, collected from literature search.

Supplemental references

1. Irizarry RA, Hobbs B, Collin F, et al. Exploration, normalization, and summaries of high density oligonucleotide array probe level data. Biostat Oxf Engl 2003;4(2):249–264.

2. Babicki S, Arndt D, Marcu A, et al. Heatmapper: web-enabled heat mapping for all. Nucleic Acids Res 2016;44(W1):W147-153.

Supplemental Figure Legends

Supplemental Figure 1. (A) Unsupervised analysis using multidimensional scaling (MDS) based on gene expression data from the CoMMpas study. 73 MM samples with del17p were selected for the analysis. Only the 35,345 genes interrogated by the HTA were considered for the analysis. **(B)** Sample classification in subgroups in the MDS from the CoMMpass dataset was performed using model-based methods from the mclust R package (v.5.4.1). The best model was chosen from a list of 14 methods according to their Bayesian Information Criterion (BIC). The best fitted model according to the BIC was a spherical multivariate normal model with one component. This model was selected as the best approximation because it is a one component generalization of the top two models, EII (spherical, equal volume) and VII (spherical, unequal volume), that fitted CoMMpass data similarly well with a BIC = -1435.9.

Supplemental Figure 2. Heatmap of the 65 differentially expressed genes identified by KEGG enrichment analysis based on gene expression. The heatmap was constructed using the freely available web tool Heatmapper.

Supplemental Figure 3. KEGG enrichment analysis based on genes with deregulated exons detected in pPCL and MM samples. Statistical significance of the enrichment is expressed as -log10 FDR using the Benjamini-Hochberg method.

Supplemental Figure 4. Validation of differential expression of exons by qRT-PCR. The results are represented as ΔCt between pPCL and MM samples, using *PGK1* as endogenous control gene. Statistically significant differences between pPCL and MM samples are represented as ***$p < 0.001$, **$p < 0.01$, *$p < 0.05$ and n.s no significant (two-sided Student's t-test for unpaired samples).

Supplemental Figure 5. (A) Gene expression analysis of *SRSF1* and *SRSF3 genes* by qRT-PCR. The results are represented as ΔCt between pPCL and MM samples, using *18S* as endogenous control gene. Statistically significant differences between pPCL and MM samples are represented as ***$p < 0.001$, **$p < 0.01$ (two-sided Student's t-test for unpaired samples). Pearson correlation of gene expression measured by qRT-PCR in pPCL and MM samples. **(B)** Pearson correlation analysis between gene expression of SR protein family based on HTAS data and the 13 validated exons. Statistical significances are represented as ***$p < 0.001$, **$p < 0.01$, *$p < 0.05$. **(C)** Pearson correlation analysis between *SRSF1* gene expression and the expression of *CD27* exon 2, *NEDD4L* exon 3 and *RNF11* exon 3. **(D)** Pearson correlation analysis between *SRSF3* gene expression and the expression of *CD27* exon 2 and *MERTK* exon 3

Supplemental Figure 6. Unsupervised analysis based on the expression of the expression of 158,625 isoforms in MM and pPCL samples using multidimensional scaling (MDS) of 19 samples (9 pPCL and 10 MM). Samples tend to be clustered according isoform expression in two groups.

Supplemental Figure 7. (A) Coding isoforms overexpressed (upper-red) and underexpressed (below-blue) from genes without total expression changes. The data is represented as the magnitude of isoform change (log2 FC) between pPCL and MM samples. The selected isoforms for experimental validation, based on higher, lower and medium FC are highlight in yellow. **(B)** Structure of the analysed isoforms (*IKZF1, SF1, KLC1, ASS1, RPTOR, IRF5, RPL10, RPL12* and *DLGAP4* genes) is highlighted in yellow. The two *IKZF1* non-coding isoforms are highlighted in blue. The protein domains are also indicates. The selected isoform for The yellow boxes Pictures were taken from the genome browser Ensembl and modified. **(C)** Differential expression of *IKZF1* isoforms detected in HTA analysis. Coding isoforms are represented by blue columns and non-coding isoforms by dark grey. The light blue column represents the coding isoform that does not have specific sequence for their identification among the all isoforms.

Supplemental Figure 8. Gene expression of SR proteins in pPCL compared to MM patients from HTA results. Columns in red, represents p-value < 0.05 and columns in pink, p-value > 0.05, for SR gene expression.

Supplemental table 1. *TP53* status

Patient	Group	*TP53* deletion (%) by FISH	*TP53* status	AA change	Mutation cDNA	Mutation allelic fraction	Mutant p53 transactivation activity
46061	MM	71	WT				
46541	MM	83	WT				
46363	MM	83	WT				
45814	MM	75	WT				
45627	MM	83	WT				
44335	MM	95	WT				
44522	MM	96	Mut	p.Y126C	c.377A>G	100%	This codon is close to the end of an exon: splicing can be altered
47803	MM	98	Mut	p.R213X	c.637C>t	100%	The activity of truncated p53 is assumed to be nil
44728	MM	93	Mut	p.C275R	c.823T>C	80%	This mutant is inactive
47405	MM	94	WT				
17012	pPCL	88	no gDNA				
22335	pPCL	93	Mut	p.H179R	c.536A>G	100%	This mutant is inactive
28262	pPCL	72	WT				
30655	pPCL	87	Mut	p.Y205D	c.613T>G	80%	This mutant is inactive
32186	pPCL	97	Mut	p.R280T	c.839G>C	100%	This mutant is inactive
47555	pPCL	97	WT				
45785	pPCL	84	WT				
48401	pPCL	20	Mut	p.L257Q	c.770T>A	50%	This mutant is inactive
20230	pPCL	89	WT				

AA: aminoacid; WT: wild type *TP53*; Mut: mutated *TP53*; no gDNA: genomic DNA no available.

Suplemental Figure 1

A

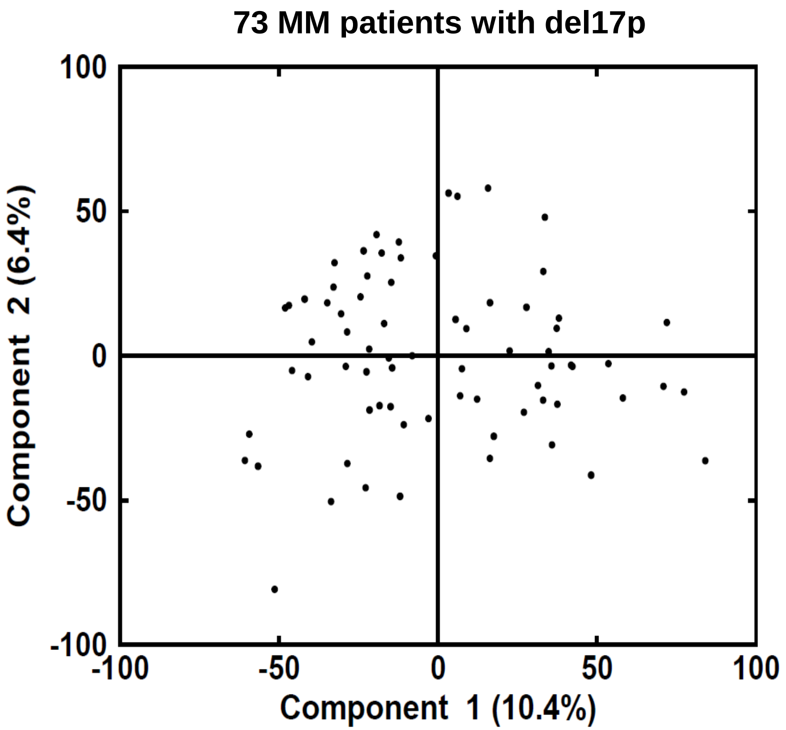

B

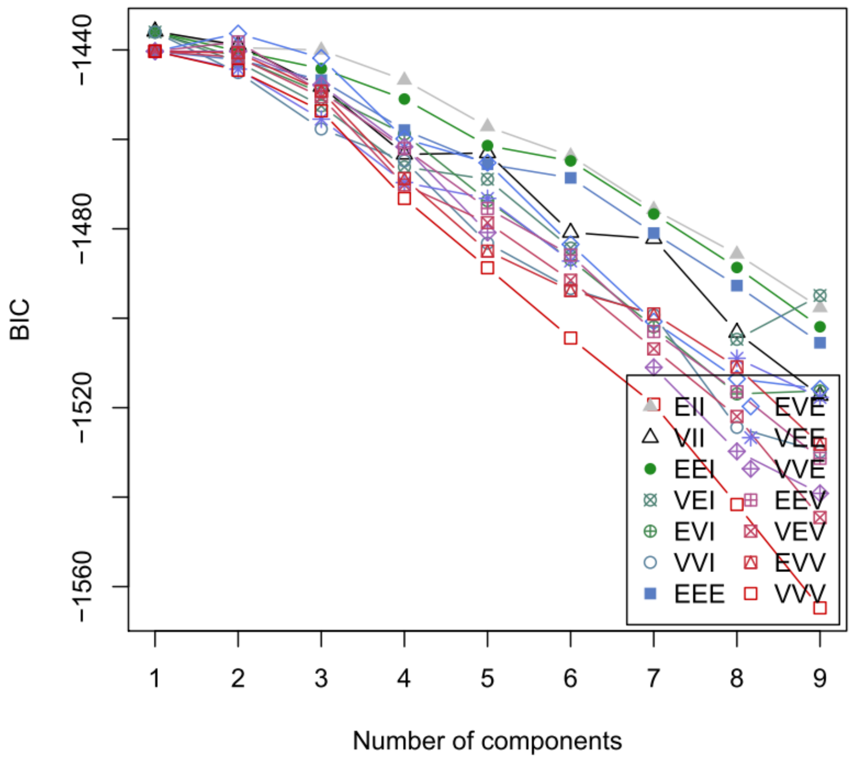

Suplemental Figure 2

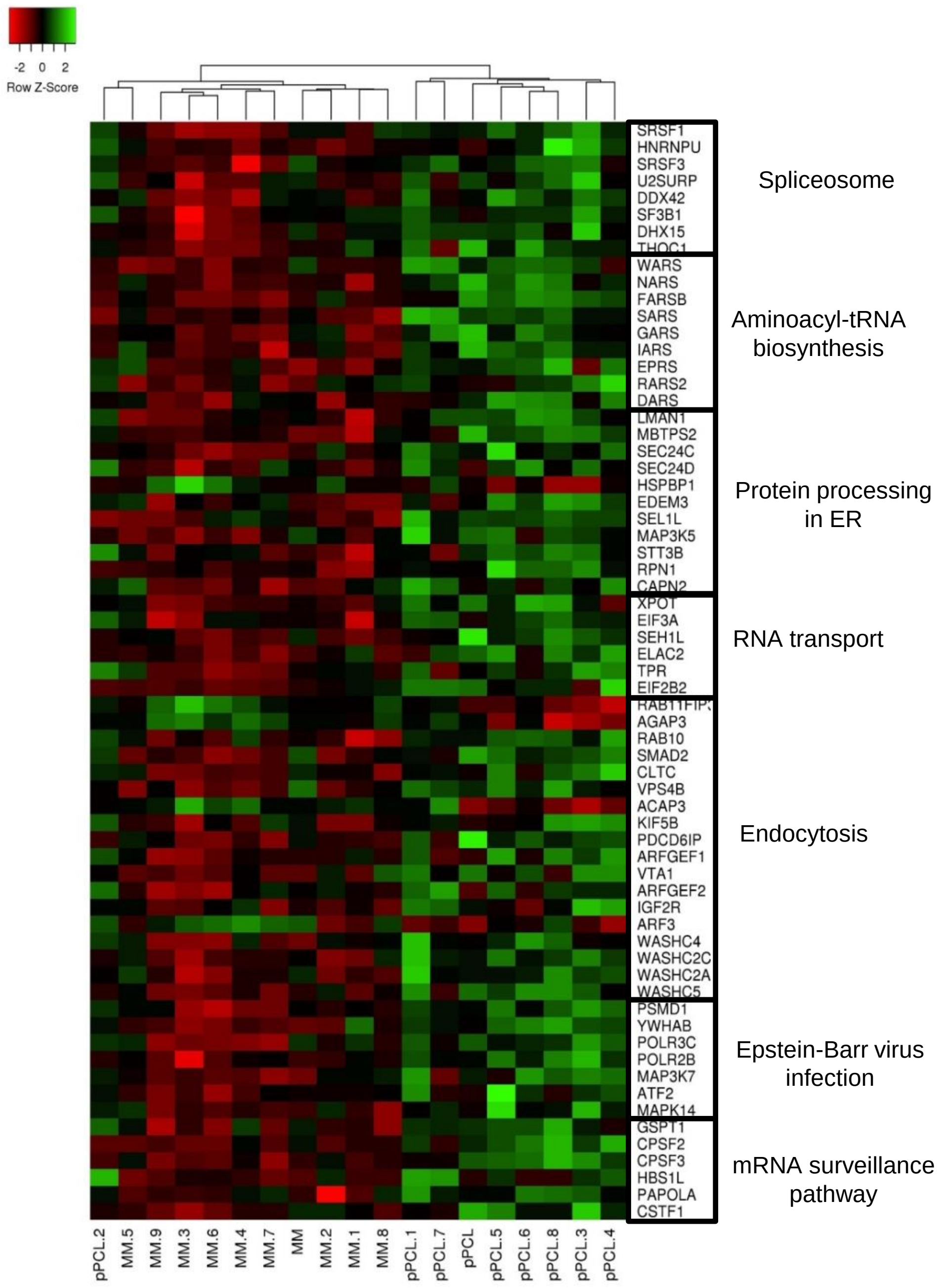

Supplemental table 2. Primers used in qRT-PCR (Taqman-based detection) GENE EXPRESSION VALIDATION

Gene	Assay ID	Catalog number
SRSF1	Hs00199471_m1	4448892
SRSF3	Hs00751507_s1	4448892
SF3B1	Hs00961640_g1	4448892
DDX42	Hs00201296_m1	4448892
DHX15	Hs00154713_m1	4448892
HNRNPU	Hs00244919_m1	4448892
THOC1	Hs01018285_m1	4448892
U2SURP	Hs01030366_m1	4448892
TP53	Hs01034249_m1	4331182
18S (endogenous control gene)	Hs99999901_s1	4331182
WARS	Hs00188259_m1	4448892
AHR	Hs00169233_m1	4453320
DUSP5	Hs00244839_m1	4453320
CD79A	Hs00998119_m1	4448892
GADD45A	Hs00169255_m1	4453320
GADD45B	Hs00169587_m1	4453320
PDE4B	Hs00277080_m1	4448892

Suplemental Figure 3

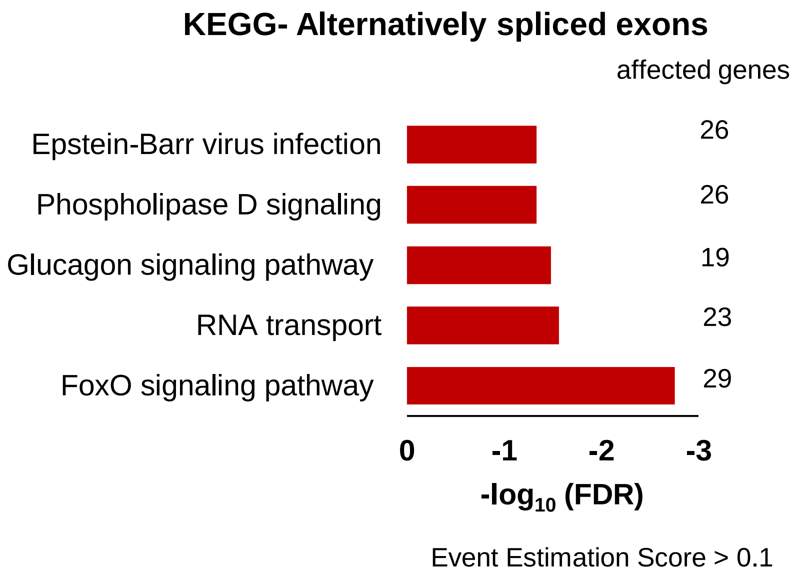

Supplemental table 3. Validation of cassette exon (CE) events

Gene	FC HTAs	FC qRT-PCR	Splicing Event Estimate
TRA2B	2.7	1.5	More exon inclusion in pPCL (CE)
TUBG1	3.1	1.6	More exon inclusion in pPCL (CE)
BCL2	2.5	3.0	More exon inclusion in pPCL (CE)
COA3	4.9	2.8	More exon inclusion in pPCL (CE)
COG8	2.7	1.7	More exon inclusion in pPCL (CE)
PI4KB	3.1	1.4	More exon inclusion in pPCL (CE)
PPM1B	3.4	2.6	More exon inclusion in pPCL (CE)
RNF11	-3.6	-2.1	More exon exclusion in pPCL (CE)
MERTK	-32.7	-2.4	More exon exclusion in pPCL (CE)
SULF2	-145.8	-9.1	More exon exclusion in pPCL (CE)
CD27	-27.6	-9.4	More exon exclusion in pPCL (CE)
NEDD4L	-27.7	-4.5	More exon exclusion in pPCL (CE)
KRAS	-2.4	-1.1	More exon exclusion in pPCL (CE)

Supplemental table 4. Primers used in qRT-PCR (SYBR Green-based detection) to CASSETTE EXON EVENTS

Gene	Oligo Name	Sequence 5' to 3'
TRA2B	TRA2B_Fw	GATGAAGCGTGAGTTTCCTGC
	TRA2B_Rv	GTGACATTGGAGTCAATCGGC
TUBG1	TUBG1_Fw	CTGTCGCCAGTATGACAAGC
	TUBG1_Rv	AGAAAGAGATGCGTGAGGTCC
BCL2	BCL2_Fw	TGCAGTTGGGCAACAGAGAA
	BCL2_Rv	ACATGTGTTGGGATTGCCCT
COA3	COA3_Fw	GCGTCAGGGTCCTAATCTGG
	COA3_Rv	CACAGAGCTGCCACGTCTAA
COG8	COG8_Fw	GCTGATTTCCGGGGTCAGTT
	COG8_Rv	GGGGTGGGAAATCTAGGAGC
PI4KB	PI4KB_Fw	TGGACAGTGAGATCCGTTGC
	PI4KB_Rv	GGGGCAGATAGAAGTCCACG
PPM1B	PPM1B_Fw	CTTGCGGATTCCCAACGTTTT
	PPM1B_Rv	ACGAGTATGCCTGAGGTTGG
RNF11	RNF11_Fw	CAATTCGATTTCTGCCGTGC
	RNF11_Rv	CAGTGCTGCATCAACTGGCT
SULF2	SULF2_E5_Fw	ACCAATGACAGCGTGAGCTT
	SULF2_E5_Rv	GCGTGAATATTGTGGGGCTG
MERTK	MERTK_E3_Fw	CATAACCAGTGTGCAGCGTTC
	MERTK_E3_Rv	ATGGGATCAGACACGATCTCTTC
NEDD4L	NEDD4L_E3_Fw	CTTCTGCTCCATTGCTGTTGA
	NEDD4L_E3_Rv	TGCTATTATCAGACTCACACTTGGT
CD27	CD27_E2_Fw	AGCATAGAAAGGCTGCTCAGT
	CD27_E2_Rv	GTGCCGACAGCTCTCACAGT
KRAS	KRAS_oE4_Fw	ACATTGGTGAGAGAGATCCGA
	KRAS_oE4_Rv	ACACAGCCAGGAGTCTTTTCT

Supplemental Figure 4

Validation of inclusion/exclusion of exon

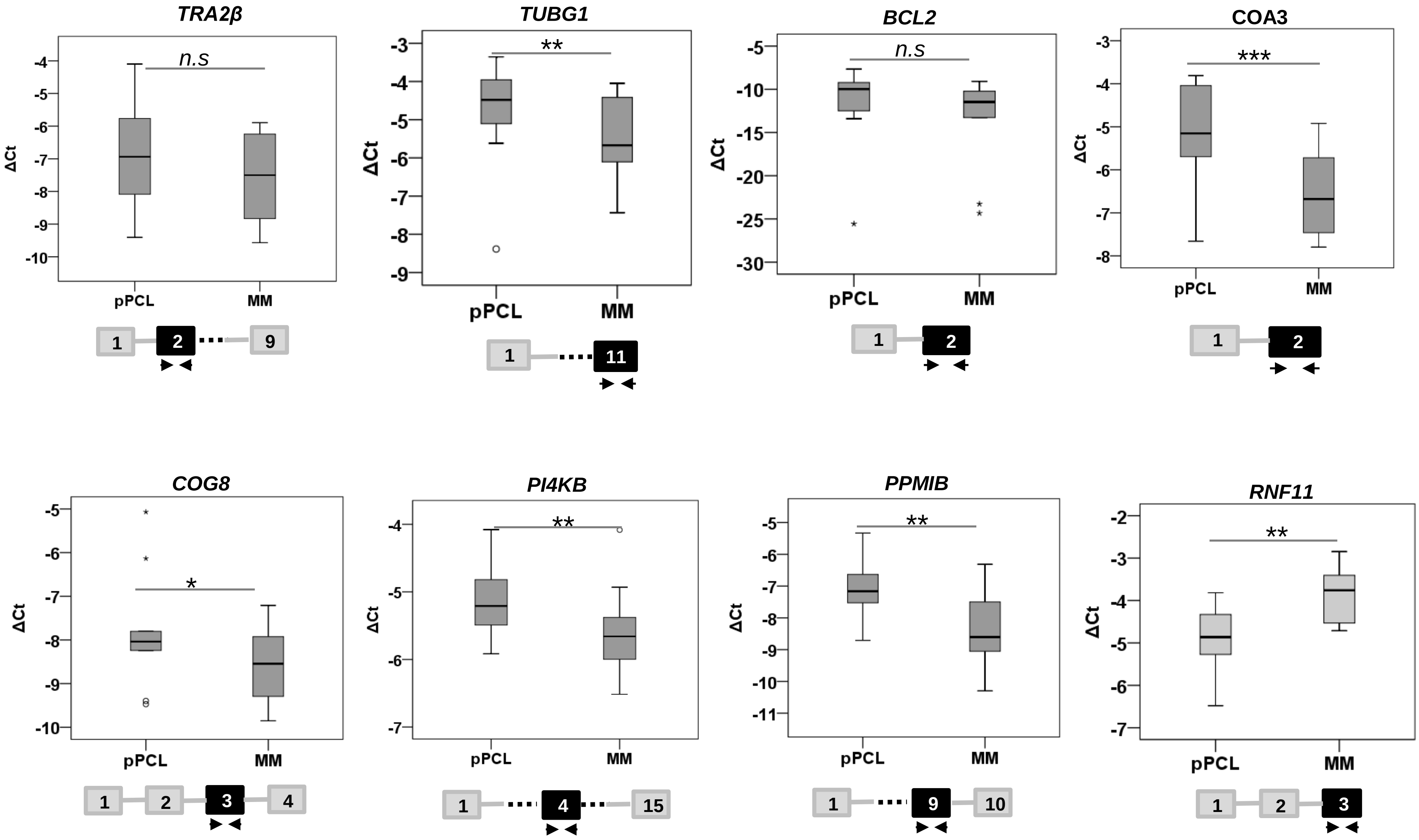

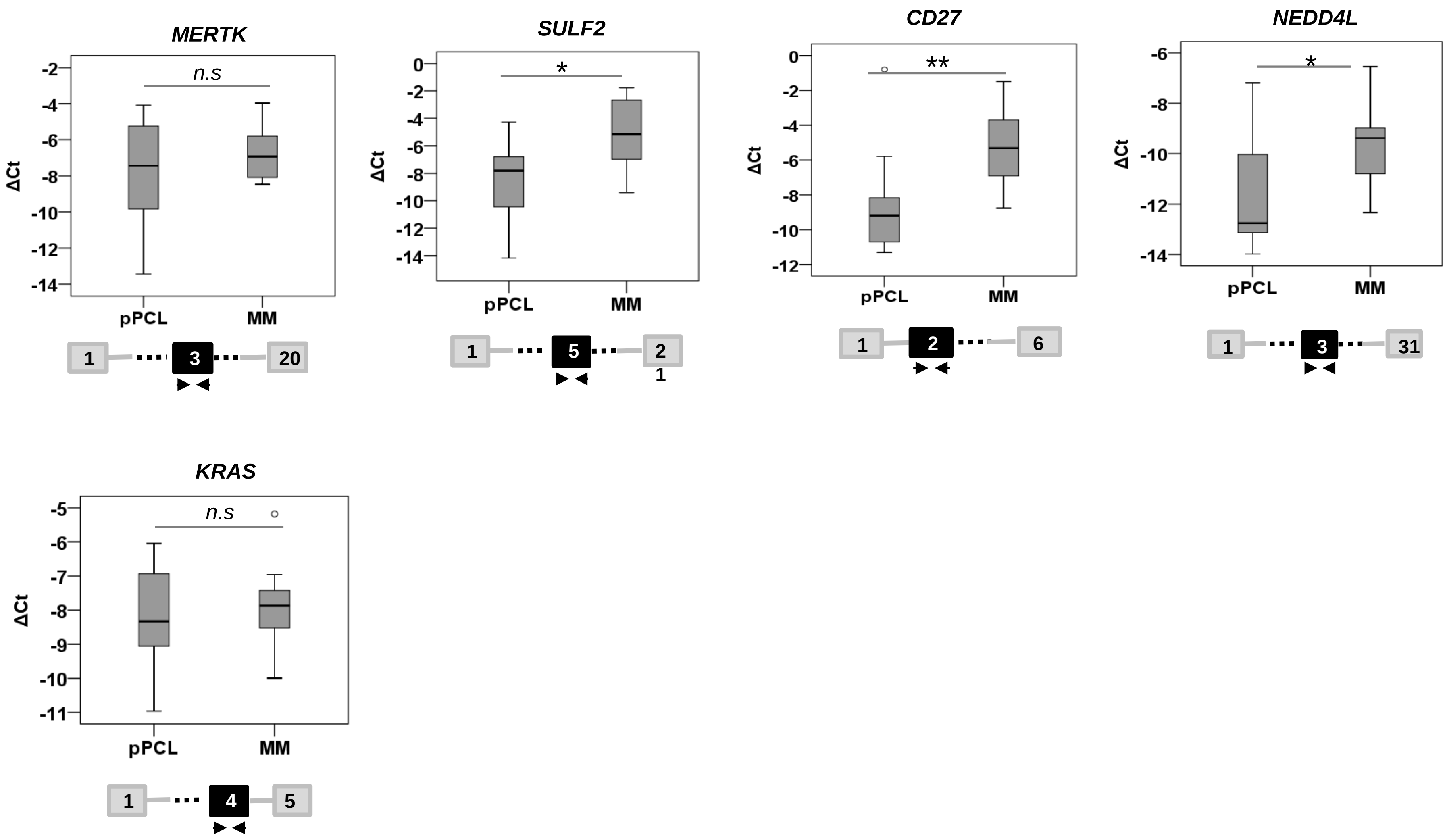
MERTK
n.s
-2
-4
-6
-8
-10
-12
-14
ΔCt
pPCL
MM
1
3
20
SULF2
*
0
-2
-4
-6
-8
-10
-12
-14
ΔCt
pPCL
MM
1
5
2
1
CD27
**
0
-2
-4
-6
-8
-10
-12
ΔCt
pPCL
MM
1
2
6
NEDD4L
*
-6
-8
-10
-12
-14
ΔCt
pPCL
MM
1
3
31
KRAS
n.s
-5
-6
-7
-8
-9
-10
-11
ΔCt
pPCL
MM
1
4
5

Supplemental Figure 5

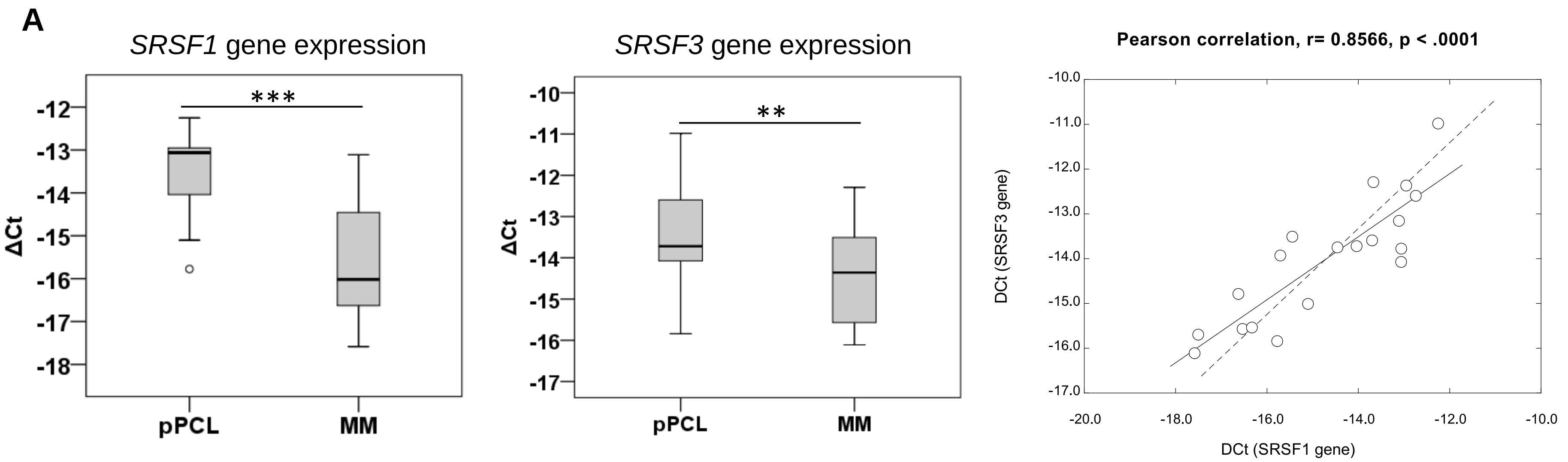

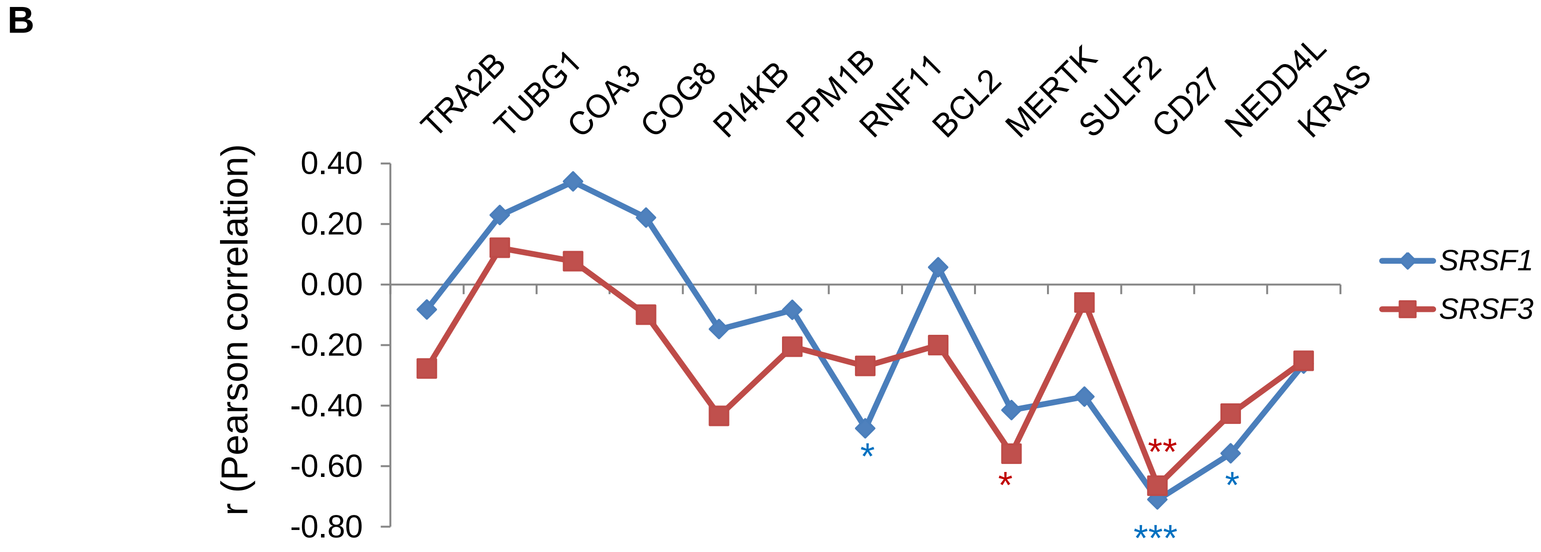

Supplemental Figure 5

C

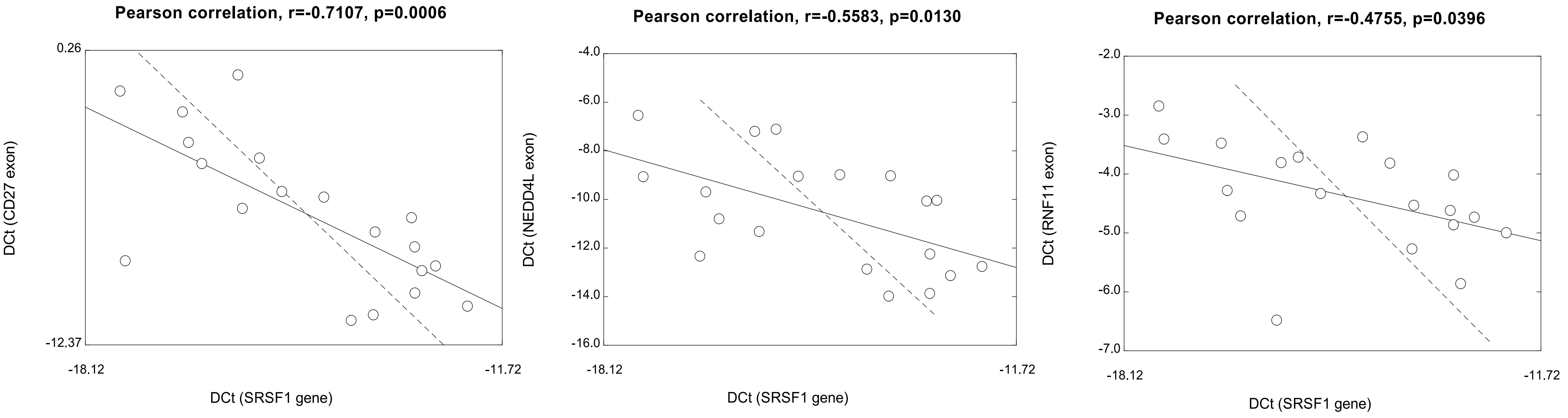

D

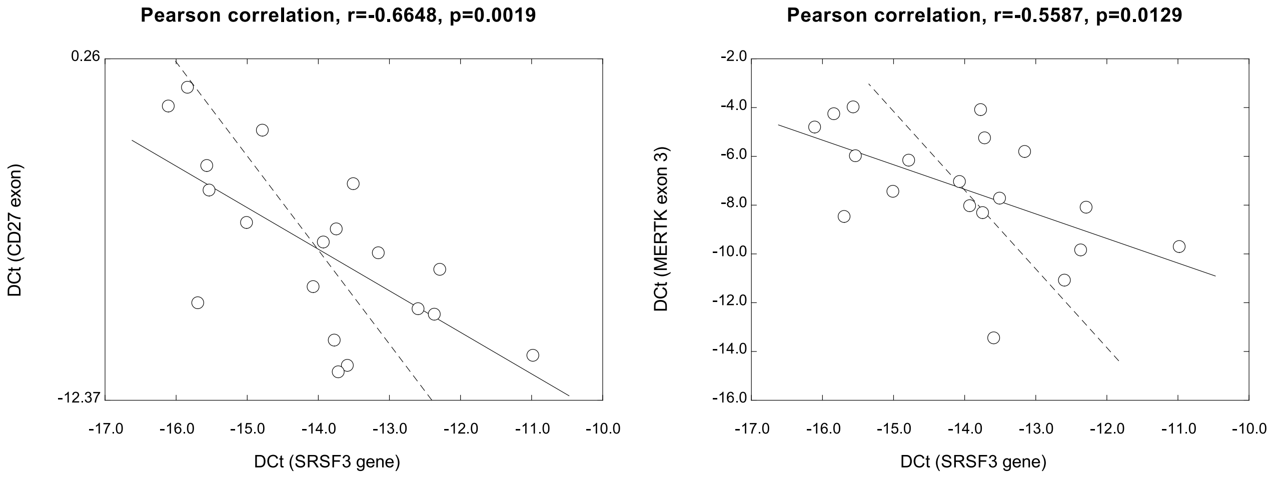

Suplemental Figure 6

Unsupervised analysis based on isoform expression

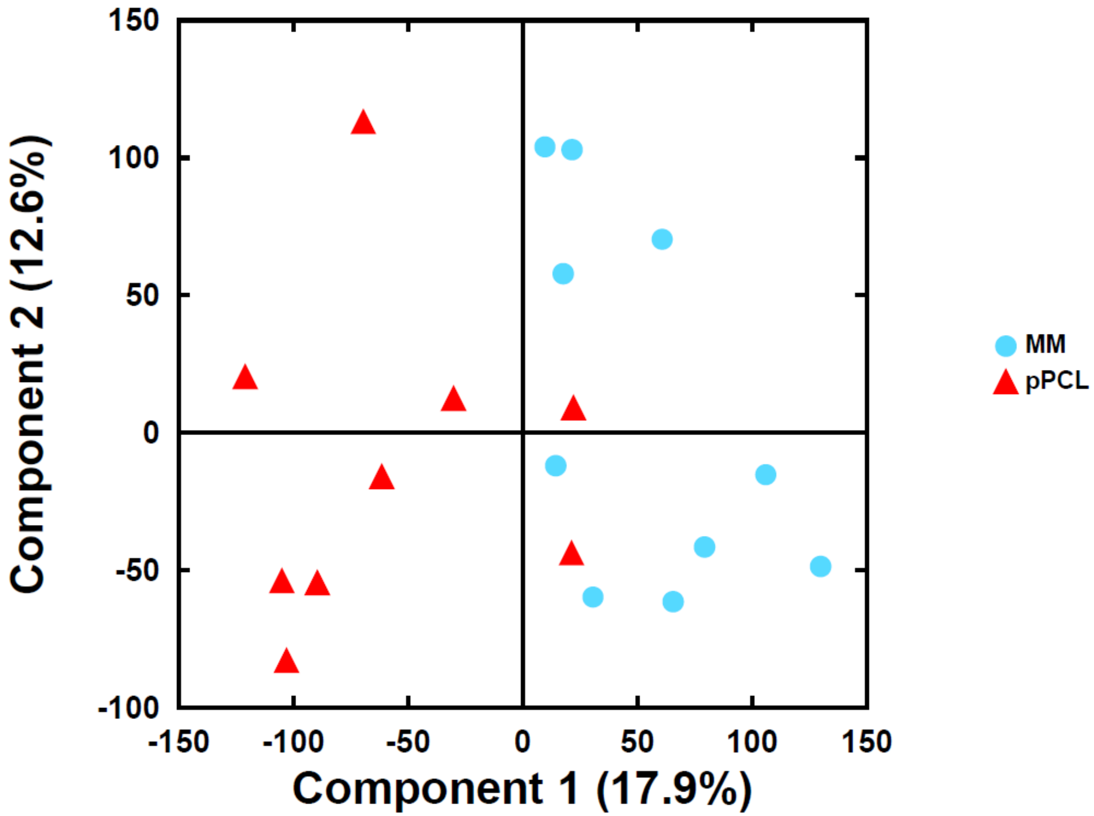

Suplemental Figure 7

A

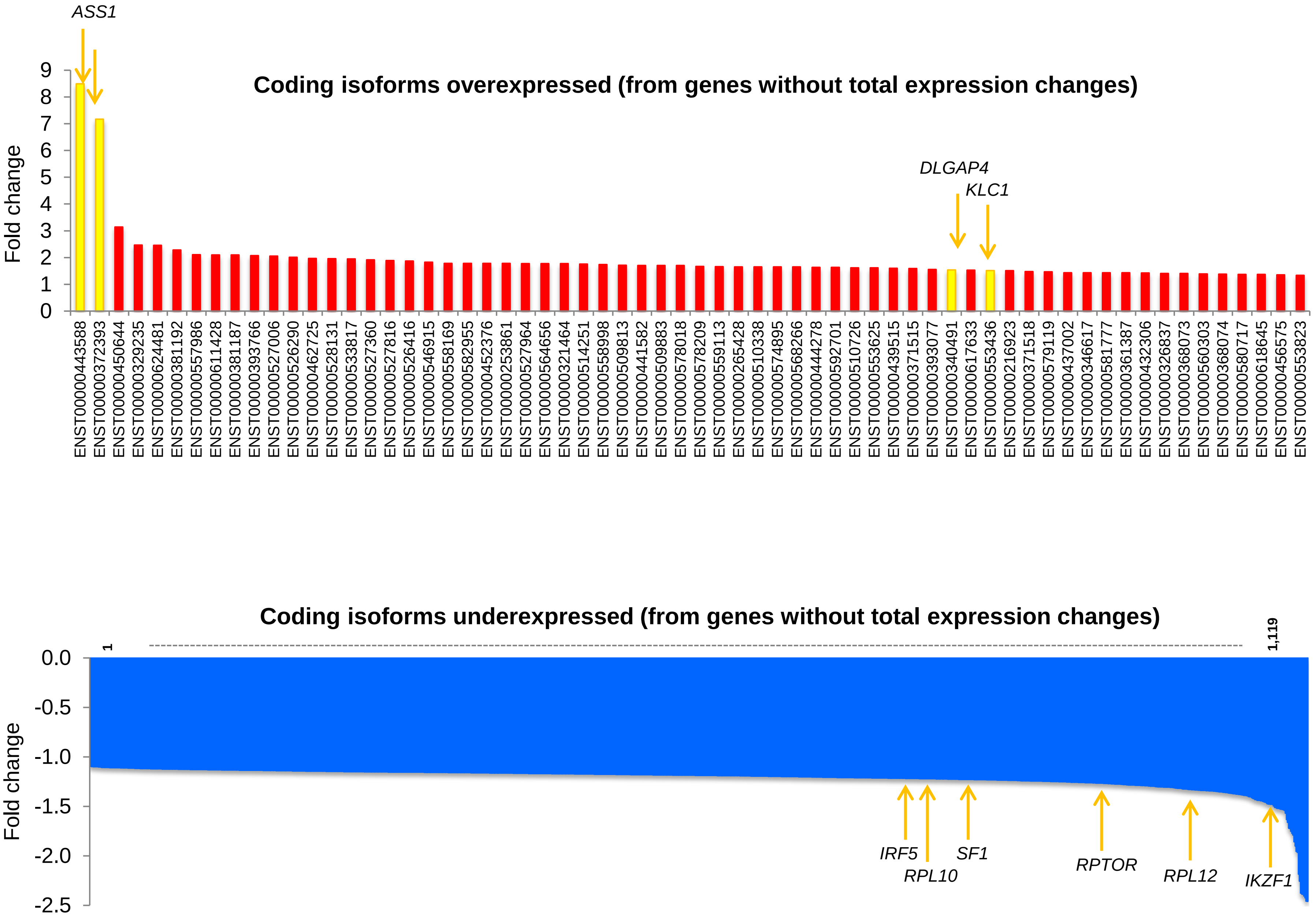

Supplemental table 5. Summary of tested deregulated coding isoforms (from genes without total expression changes)

HTA

GENE				ISOFORM		
**HGNC**	**Ensembl ID**	**FC**	***q*-value**	**Isoform**	**Fold Change**	***q*-value**
RPTOR	ENSG00000141564	1.0903	0.204	ENST00000576366	0.787	0.005
RPL10	ENSG00000147403	0.9421	0.204	ENST00000449494	0.817	0.005
IRF5	ENSG00000128604	0.9122	0.062	ENST00000464557	0.820	0.005
IKZF1	ENSG00000185811	0.8126	0.075	ENST00000413698	0.676	0.016
IKZF1	ENSG00000185811	0.8126	0.075	ENST00000646110	0.669	0.021
IKZF1	ENSG00000185811	0.8126	0.075	ENST00000612658	0.692	0.046
RPL12	ENSG00000197958	0.9923	0.374	ENST00000536368	0.745	0.028
DLGAP4	ENSG00000080845	1.0600	0.204	ENST00000340491	1.541	0.036
SF1	ENSG00000168066	1.0045	0.425	ENST00000413725	0.815	0.046
KLC1	ENSG00000126214	1.0131	0.420	ENST00000553436	1.518	0.046
ASS1	ENSG00000130707	1.0251	0.414	ENST00000372393	7.174	0.046
ASS1	ENSG00000130707	1.0251	0.414	ENST00000443588	8.497	0.046

Supplemental table 6. Primers used in qRT-PCR (SYBR Green-based detection) to ISOFORM VALIDATION

Gene	Isoform (ensembl anotation)	Oligo Name	Sequence 5' to 3'
IKZF1	ENST00000413698	IKZF1_698_Fw	CGTACGTGCATGTTCCTTCATC
		IKZF1_698_Rv	TGTTACTCCACTGCCATGCT
	ENST00000646110	IKZF1_110_Fw	AGCAGGACACTCTAACAAGTGAC
		IKZF1_110_Rv	GCATCAAGACCAAGTAGCCG
	ENST00000612658	IKZF1_658_Fw	ACCCGAGGATCAGTCTTGG
		IKZF1_658_Rv	GTTGCCCTTCTGGGTGAAT
SF1	ENST00000413725	SF1_Fw	GCCCTCCTCCAATGGGTAAA
		SF1_Rv	CTAGTTCTGTGGTGGAGGCG
RPL10	ENST00000449494	RPL10_ENS494_Fw	CTAGAGACACAGCAGCCATCA
		RPL10_ENS494_Rv	TAGAGAAGGAGCTGAACGCC
RPL12	ENST00000536368	RPL12_Fw	CTGAGGTGCACCGGAGGT
		RPL12_Rv	TGTTTTTCTGTTTCTTTCTGTCTCTT
KLC1	ENST00000553436	KLC1_Fw	TGAGAACATGGAGAAGCGCA
		KLC1_Rv	TCTAGCACATGAGACAGGAGGA
IRF5	ENST00000464557	IRF5_Fw	TCCTAAGTGCTACCCGAATGC
		IRF5_Rv	ACCTCTCTAGGGCCATTCATCT
DLGAP4	ENST00000340491	DLGAP4_Fw	TGTCTCTTTGTCTCTGCCCTC
		DLGAP4_Rv	AGAACCCCCAAAGGATGTGC
RPTOR	ENST00000576366	RPTOR_Fw	GACACGGAAGATGTTCGACAAG
		RPTOR_Rv	GAAGCAACGCTCTCAGTGT
ASS1	ENST00000372393	ASS1_393_Fw	CAGCGCACTGTATGAGGACC
		ASS1_393_Rv	CAGCTGAGCTCAAACCGGA
	ENST00000443588	ASS1_588_Fw	CAGTCCAGCGCACTGTATGA
		ASS1_588_Rv	GGGAGCAATGACCTTTCCTGT

Supplemental Figure 7

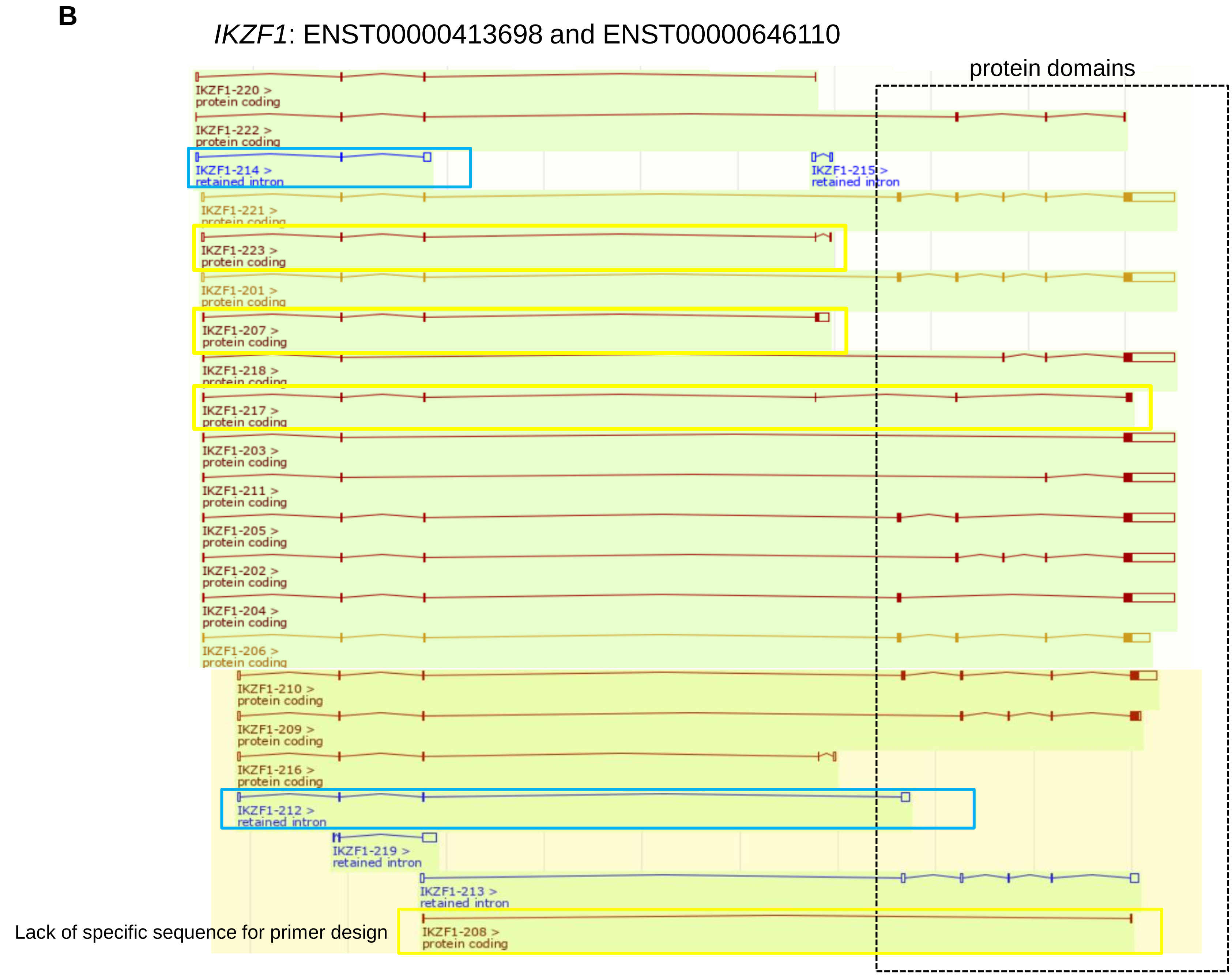

SF1: ENST00000413725

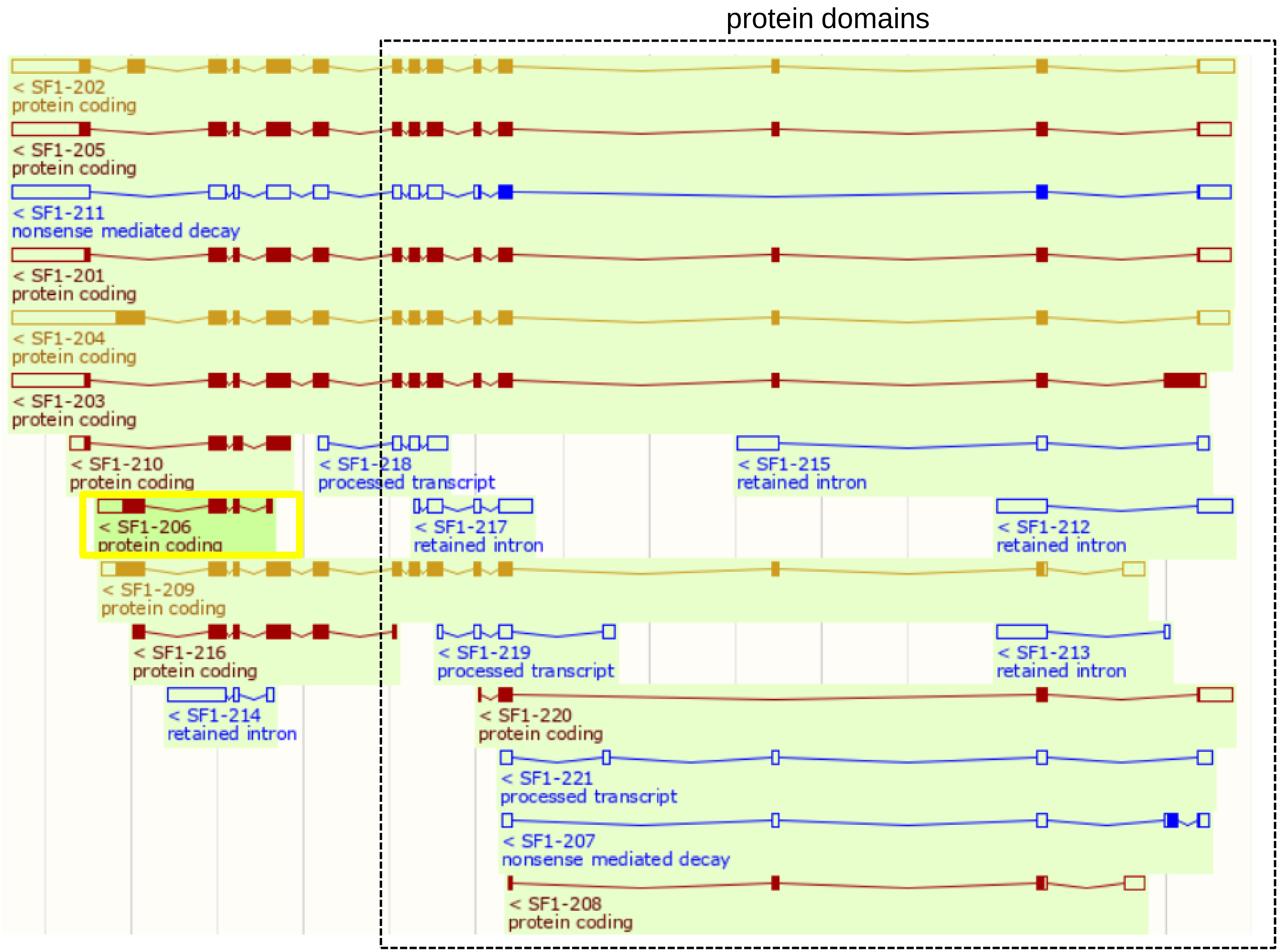

KLC1:ENST00000553436

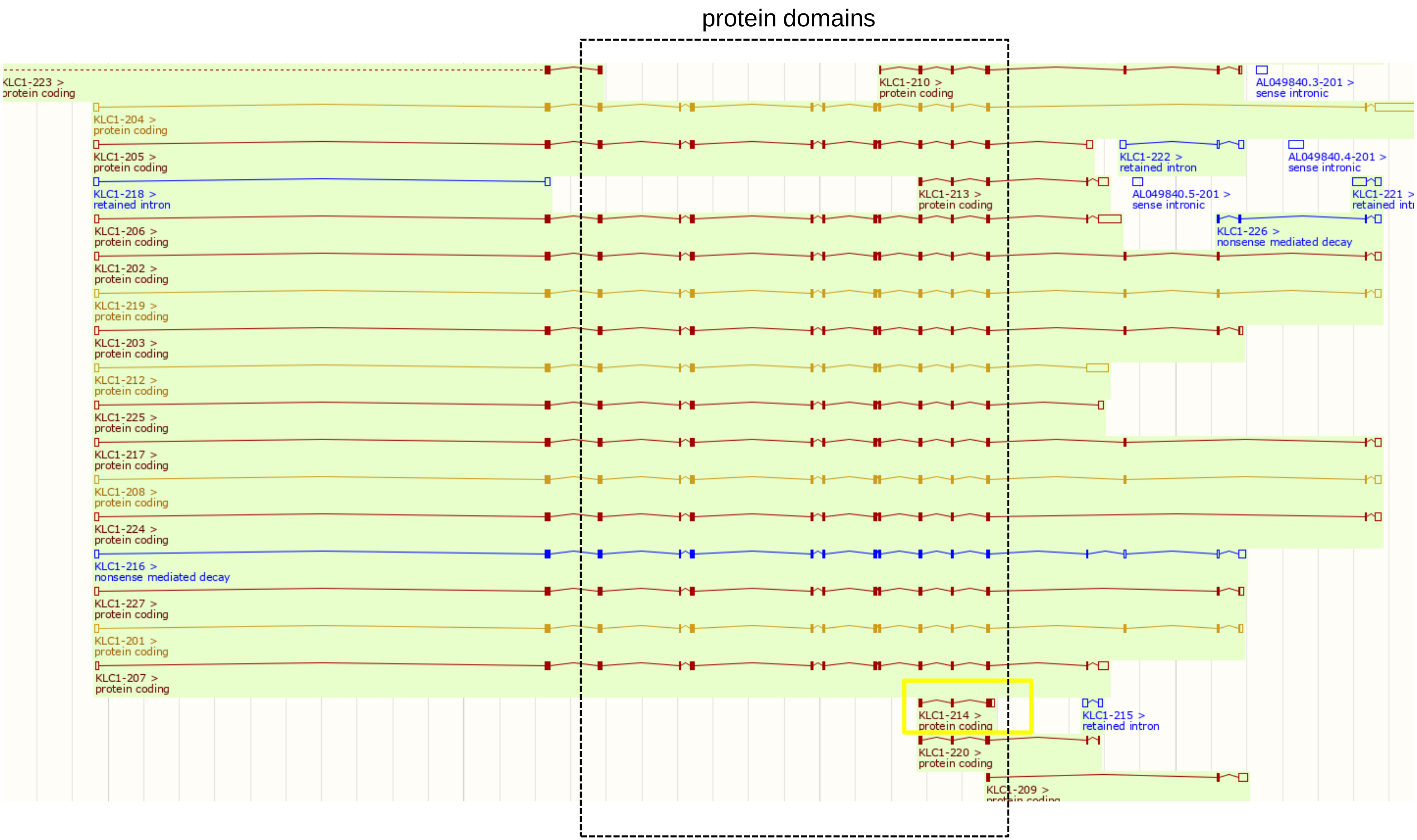

ASS1: ENST00000443588 and ENST00000372393

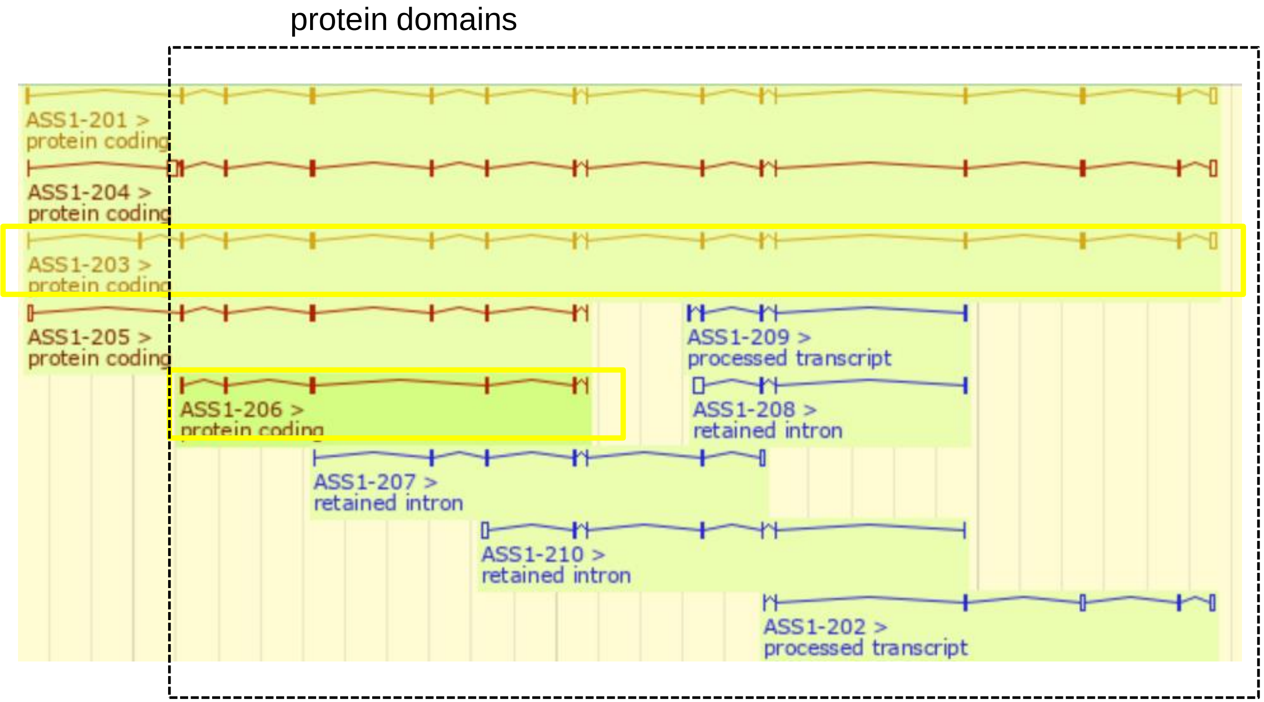

RPTOR: ENST00000576366

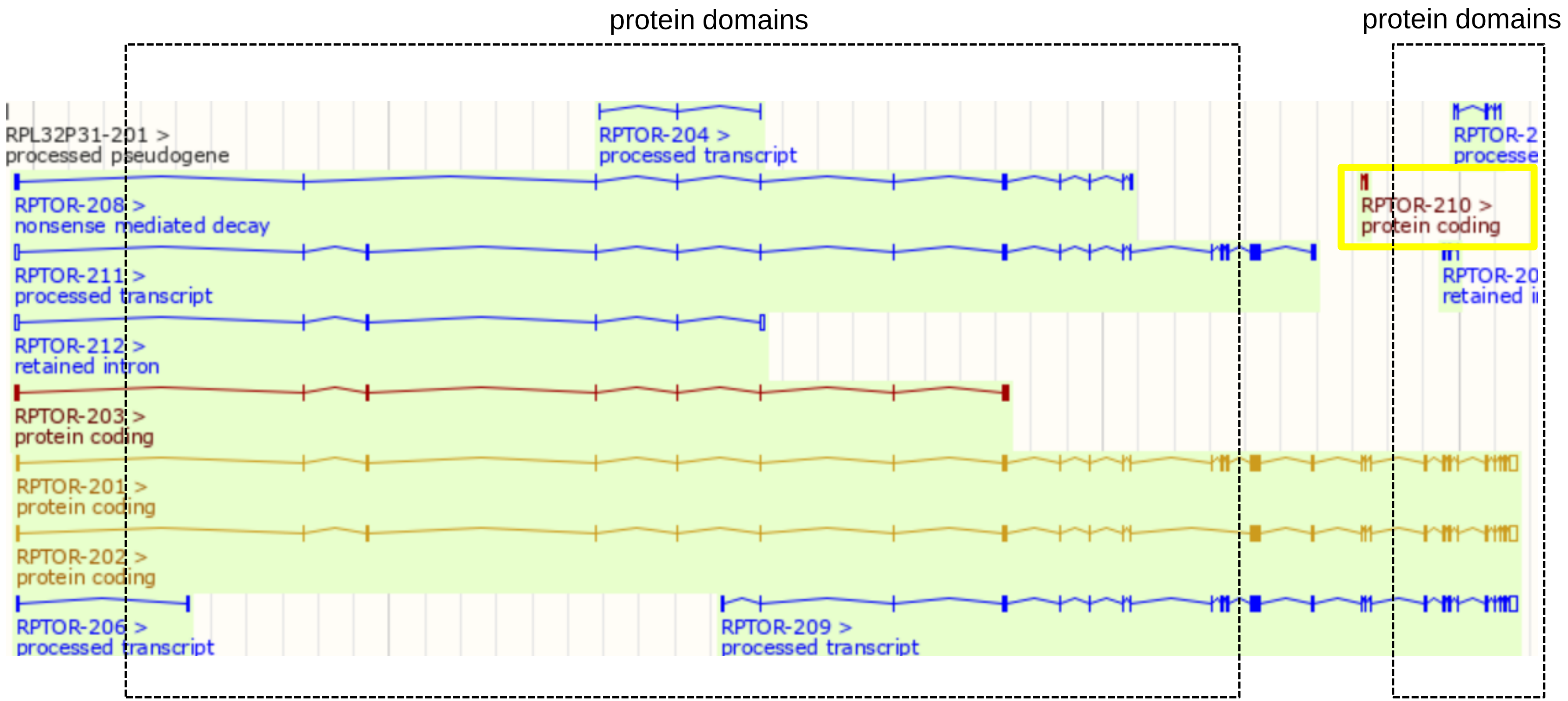

IRF5: ENST00000464557

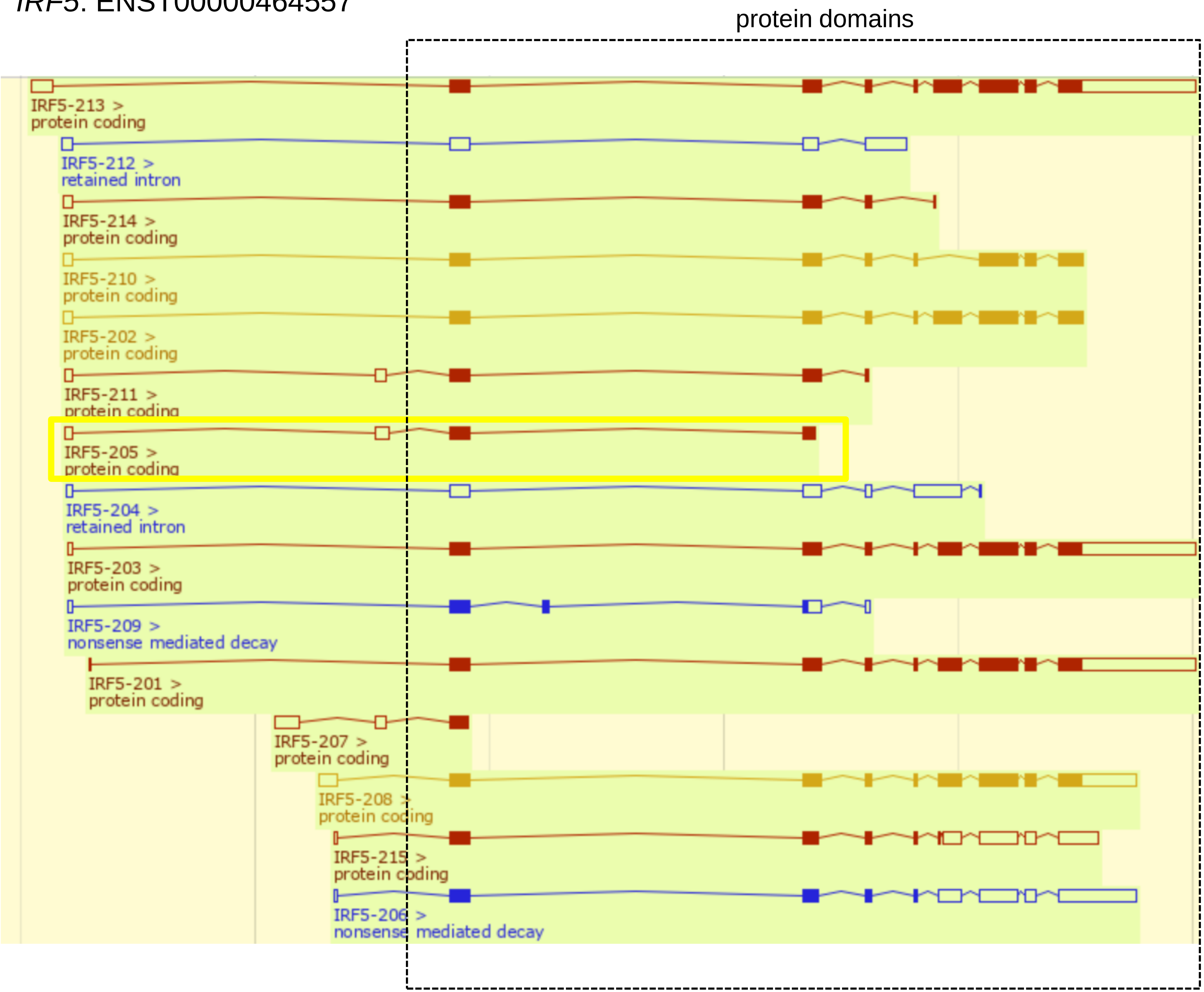

RPL10: ENST00000449494

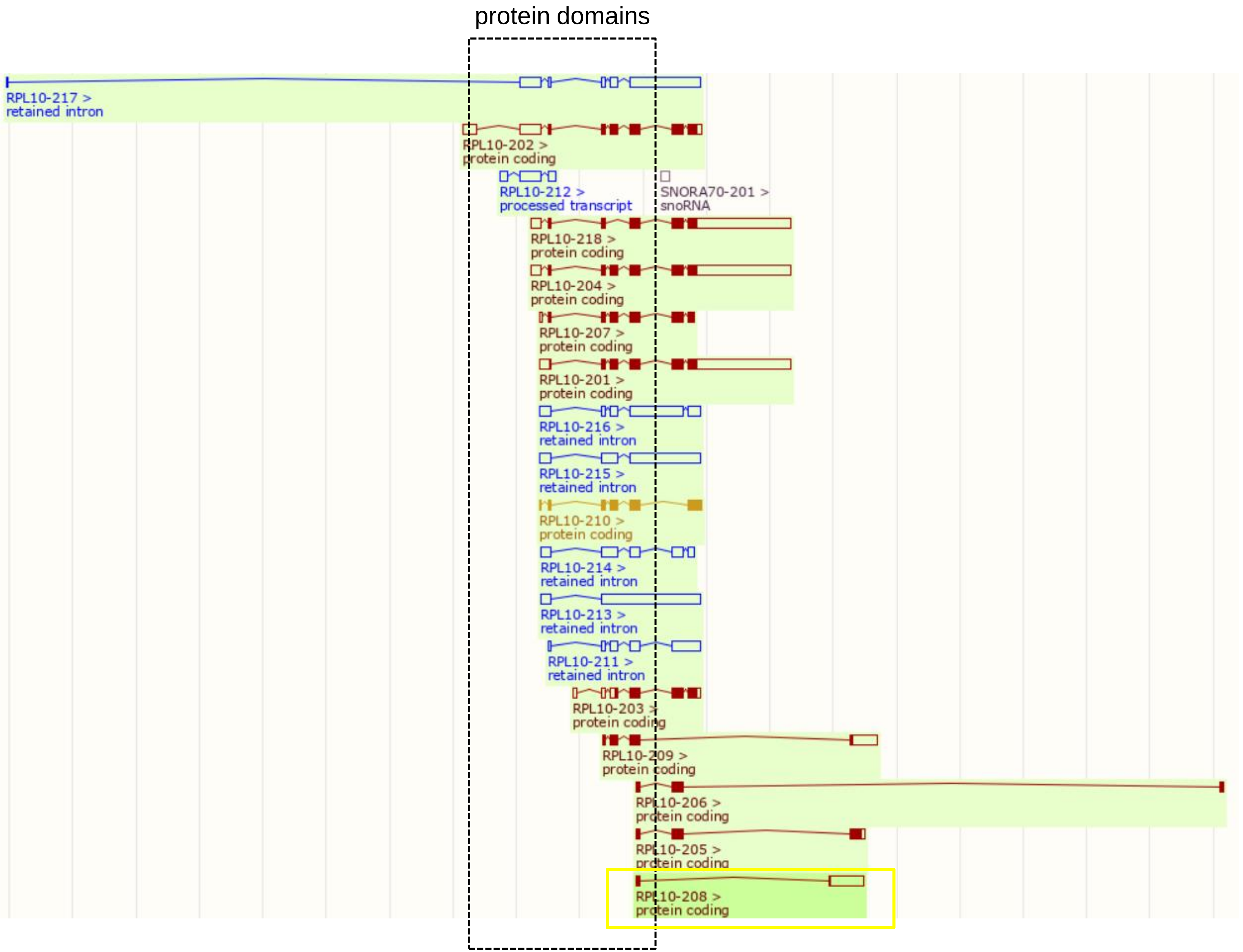

RPL12: ENST00000536368

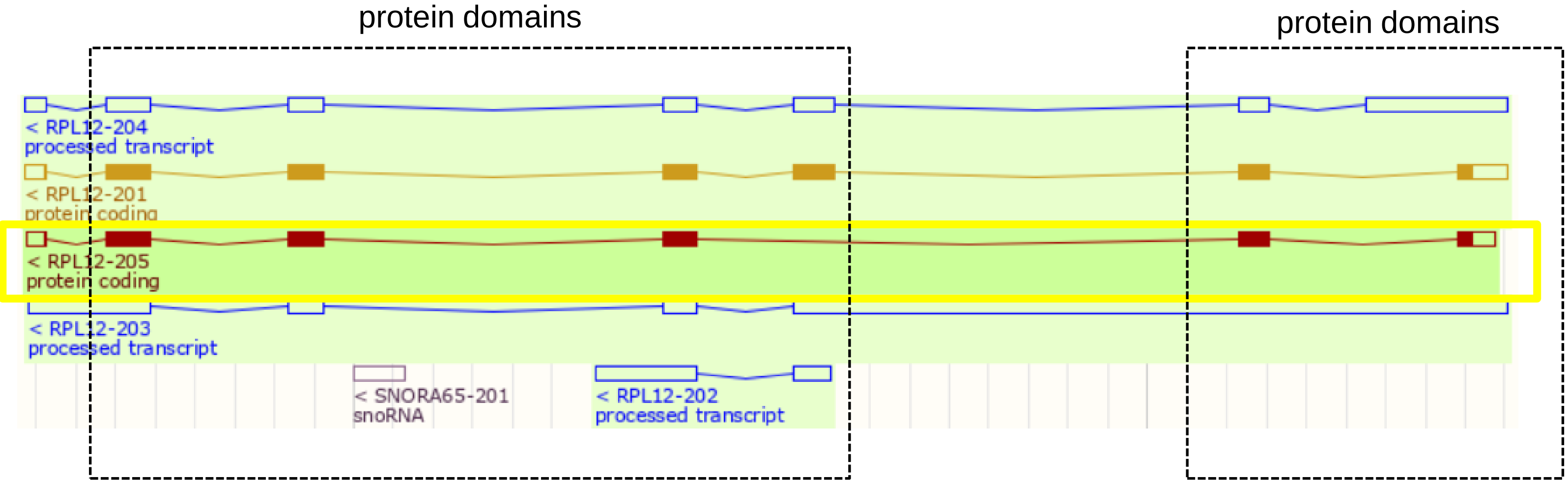

DLGAP4: ENST00000340491

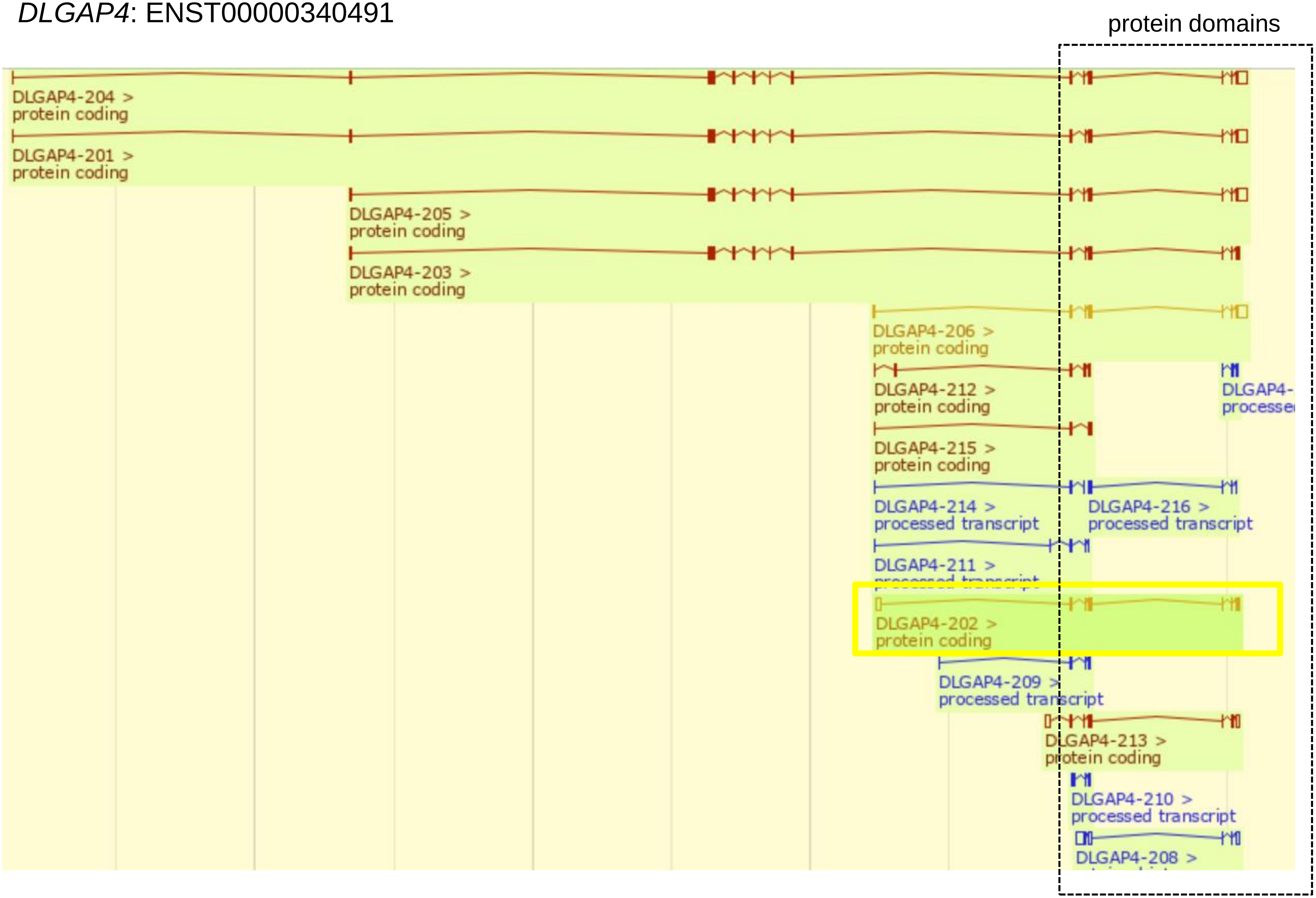

Supplemental Figure 7

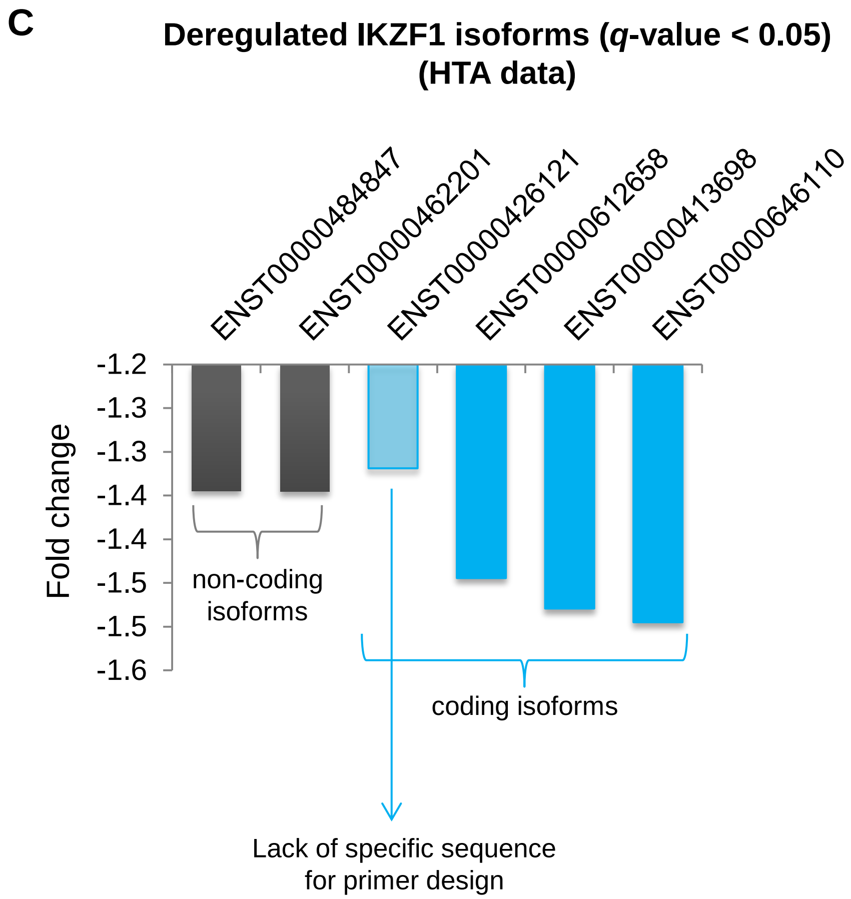

Supplemental Figure 8

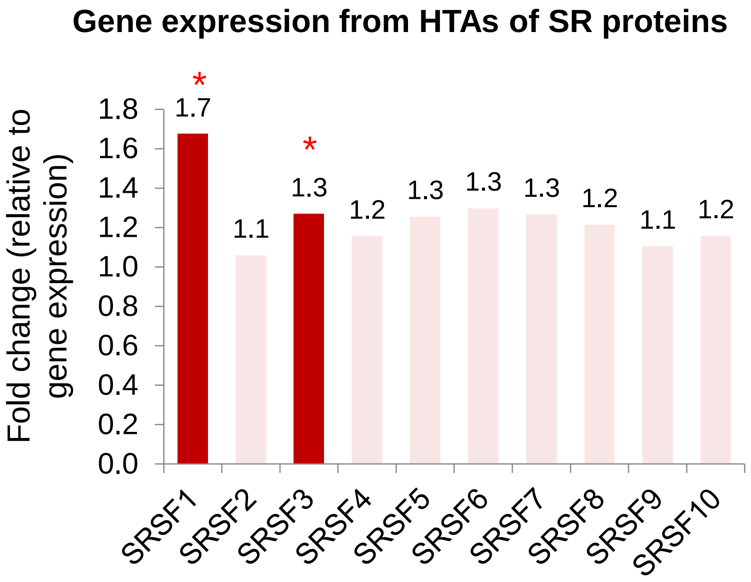

Supplemental table 6. Pathways affected by inclusion or exclusion of exons

Pathway	No. of affected genes	FDR	Genes
**FoxO signaling pathway**	26	0.00175	*PLK2, PLK3, MAPK14, AKT1, GABARAPL1, FBXO25, IKBKB, ARAF, KRAS, SMAD2, SMAD3, ATM, PCK2, PIK3CB, PIK3R1, PRKAB2, PRKAG1, BCL6, SGK1, SOS2, BRAF, TGFB2, TGFBR1, SETD7, HOMER2, HOMER1*
**RNA transport**	26	0.02720	*SNUPN, SRRM1, RPP40, STRAP, DDX20, XPOT, NUP35, NUP188, GEMIN5, UPF2, RGPD4, NUP98, ELAC1, NUP133, NUP107, XPO5, RANBP2, RGPD3, DDX39B, RAE1, THOC5, EIF3J, EIF2B4, EIF2B5, RBM8A, THOC1*
**Glucagon signaling pathway**	19	0.03300	*ATF2, AKT1, PLCB1, ACACA, ITPR1, PCK2, PDHB, PFKL, PHKA2, PHKB, PHKG2, PLCB2, PLCB3, PPP3CB, PRKAB2, PRKAG1, PPP4R3B, CREB3L2, CAMK2D*
**Phospholipase D signaling pathway**	23	0.04650	*ADCY3, RAPGEF4, DGKQ, DNM2, AKT1, PTK2B, PLCB1, PLA2G4D, GRM5, KRAS, PIK3CB, PIK3R1, PLCB2, PLCB3, PLCG2, AGPAT5, RALGDS, SHC1, SOS2, TSC1, PIP5K1A, PLPP1, CYTH1*
**Epstein-Barr virus infection**	29	0.04650	*HDAC5, POLR3A, RBPJL, ATF2, MAPK14, AKT1, DDX58 GTF2E2, HDAC1, HLA-F, HLA-G, HSPA2, IKBKB, IRF3, ITGAL, PIK3CB, PIK3R1, PLCG2, POLR3B, PSMD4, PSMD13, MAP2K4, POLR3D, TBP, TNFAIP3, POLR1C, CD44, NCOR2, HDAC4*
